# Larger wind turbines as a solution to reduce environmental impacts

**DOI:** 10.1038/s41598-024-56731-w

**Published:** 2024-03-19

**Authors:** Naveed Akhtar, Beate Geyer, Corinna Schrum

**Affiliations:** 1 Institute of Coastal Systems - Analysis and Modeling, Helmholtz-Zentrum Hereon, Geesthacht, Germany; 2 Center for Earth System Research and Sustainability, Institute of Oceanography, University of Hamburg, Hamburg, Germany

**Keywords:** Physical oceanography, Wind energy

## Abstract

The EU aims for carbon neutrality by 2050, focusing on offshore wind energy. Investments in North Sea wind farms, with optimal wind resources, play a crucial role. We employed a high-resolution regional climate model, which incorporates a wind farm parametrization, to investigate and address potential mitigating impacts of large wind farms on power generation and air-sea fluxes. Specifically, we examined the effects of replacing 5 MW turbines with larger 15 MW turbines while maintaining total capacity. Our study found that substituting 15 MW turbines increases the capacity factor by 2–3%, enhancing efficiency. However, these turbines exhibit a slightly smaller impact on 10 m wind speed (1.2–1.5%) and near-surface kinetic energy (0.1–0.2%), leading to reduced effects on sea surface heat fluxes compared to 5 MW turbines. This was confirmed by a stronger reduction in net heat flux of about 0.6–1.3% in simulations with 5 MW compared to 15 MW wind turbines. Air-sea fluxes influence ocean dynamics and marine ecosystems; therefore, minimizing these impacts is crucial. Overall, deploying 15 MW turbines in offshore wind farms may offer advantages for ocean dynamics and marine ecosystems, supporting the EU's carbon–neutral objectives.

## Introduction

The deployment of wind energy is a significant step towards reducing carbon emissions and increasing the use of renewable energy sources. Offshore wind farms (OWFs) have become a major focus in recent times due to the higher, more consistent, and reliable sea winds they offer compared to land-based wind farms^1,2^. Wind speeds over the sea, approximately 10 km off the coast, are typically 25% higher than those over land. Offshore wind resources have the capacity to generate electricity for 2–3 times longer periods compared to onshore wind resources^3,4^. Offshore wind turbines are situated far away from public areas, which makes the issue of noise, which often leads to complaints with onshore wind developments, less of a concern. Furthermore, offshore wind energy developments enable the construction of large, clustered wind farms with taller and larger wind turbines, which is not feasible on land.

The North Sea is a major hub for offshore wind energy^5^. This is due to several unique characteristics possessed by the region. Such as strong, consistent, and reliable wind resources at shallow water depth that allow bottom fixed wind turbines and other technologies to be deployed far from the shore. Europe has made significant strides in wind energy, with 207 GW of onshore and 28 GW of offshore capacity, resulting in a total installed wind power capacity of 235 GW^6^. The European Union (EU) aims to increase its offshore wind energy capacity to 60 GW by 2030 and 300 GW by 2050^7^. To support these targets, the North Seas Energy Cooperation (NSEC) has committed to achieve at least 260 GW of offshore wind capacity by 2050, which is more than 85% of the EU's offshore wind capacity target^8^. The deployment of offshore wind installations in the North Sea is expected to increase significantly in the future^9^.

Efforts to maximize power generation from offshore wind energy have led to the development of more efficient and larger wind turbines. These larger turbines have greater rotor diameters, allowing them to capture more wind and generate more electricity. Additionally, taller turbines can produce more energy due to the faster and more consistent winds found at higher altitudes, resulting in a more stable and reliable source of energy. They can also continue to operate at lower wind speeds, increasing the number of hours they can generate electricity. Moreover, larger turbines can help to reduce the number of turbines required for a given wind farm capacity, ultimately reducing the overall cost of energy production. For example, the Haliade-X 13/14 MW offshore wind turbines, with longer blades and larger rotor areas, are scheduled to be installed in the Dogger Bank, one of the world's largest offshore wind farms^10,11^ and these turbines are more efficient and less sensitive to wind speed variability, with a capacity factor (CF) ranging between 60 and 64%.

Offshore wind farms (OWFs) are typically clustered together in order to take advantage of the best wind resources and to minimize infrastructure and operating costs. However, this clustering can result in a decrease in power generation for downwind wind farms due to wakes generated by upwind wind farms. Wind turbines create wakes—areas of reduced wind speed and increased turbulence—as they extract kinetic energy from the wind to convert some of it into electrical energy and dissipate the rest as turbulent kinetic energy (TKE). The amount of TKE generated by turbines varies with the wind speed and is responsible for the formation of wakes and a downwind wind speed deficit^12–15^. These wakes can reduce the efficiency of downwind turbines by decreasing the wind speed and changing the wind direction, leading to a loss of power generation^15–18^. It is expected that wakes generated by wind farms would be longer over the ocean than over land due to the weaker turbulence intensity over the ocean^19,20^. Observational evidence suggests that wakes generated by wind farms can extend up to 50–70 km under stable stratified atmospheric conditions^21^. There is some evidence that the wakes generated by wind farms can have an impact on the local microclimate. For example, it has been observed that there is an increase in temperature by 0.5 K and humidity by 0.5 g per kilogram in wakes up to 60 km downwind of the wind farms at hub height^22^.

It's noteworthy that the majority of studies on wake dynamics have focused on either single wind turbines^23,24^ or individual wind farms^22,25–30^. Limited research exists on the analysis of wakes generated by one wind farm affecting another wind farm^16,18^. In our previous studies^14,15^, we pioneered a multi-year period basin-wide scale simulation of extensive clusters of offshore wind farms in the North Sea, encompassing both operational and planned installations. Our analysis delved into their influence on power generation and regional climate by modifying sea surface fluxes. It was found that annual wind speed deficits at hub height within the wind farms can reach 2–2.5 ms^–1^, depended on the shape of the wind farms^15^. The substantial size of wind farms and their proximity impacts not just the performance of their downwind turbines but also that of neighboring farms, resulting in a reduction of the capacity factor by 20% or more. This decrease in efficiency elevates energy production costs, leading to economic losses. Wind turbines do not only affect the wind speed within the rotor area but also reduce the surface wind speed by approximately 1.0 ms^–1^. This decrease in the annual mean wind speed results in a reduction in the annual mean values of net heat flux, indicating a 2% less heating of the atmosphere from the sea surface^14^. Further investigation revealed that the wakes generated by wind farms induce significant alterations in annual biomass primary production, causing local changes of up to ± 10%, not only within the wind farm clusters but also distributed across a broader region in the North Sea^31^.

These studies demonstrate that large offshore wind farms can exert a significant impact on the local climate and ecosystem of the North Sea. Therefore, more meticulous planning is essential to mitigate the effects of large offshore wind farms on the local climate and the ecosystem of the North Sea.

The size of the wake in a wind farm is influenced by several critical factors, including wind speed, turbine dimensions, and spacing. The management of these factors plays a pivotal role in shaping both the operational efficiency and the ecological footprint of wind farms. To mitigate the impact of wakes on power generation, one promising strategy involves upscaling wind turbines, both in terms of their height and rotor diameter, while simultaneously reducing the density of turbines, without compromising the total installed capacity. The emergence of floating wind turbines represents a concept with numerous environmental and deployment advantages. Floating turbines can be situated in deep waters, far from coastal areas, where the wind is consistently stronger and more reliable. Additionally, they offer greater flexibility for relocation and maintenance. The disparities in wakes produced by fixed and floating wind turbines are marginal, especially noticeable in higher wind speeds and lower wave heights^32^.

The deployment of larger and taller wind turbines holds the potential to significantly boost power production, particularly because wind speeds tend to be higher at greater altitudes. However, it's crucial to adopt a balanced perspective that accounts for the potential environmental and ecological consequences associated with larger turbines and lower turbine density. Striking the right equilibrium between energy output and environmental sustainability demands careful evaluation and consideration.

This research endeavors to explore the potential positive contributions of larger and taller offshore wind farms (OWFs) toward the mitigation of environmental impacts. Within the scope of this study, we investigated scenarios featuring homogenous wind farms, equipped with two distinct turbine types with varying capacities, either 5 MW or 15 MW, but with equivalent installed power across the domain. The wind farm parametrization considers these wind turbines as fixed to the bottom. The selection of 5 MW and 15 MW turbines was primarily motivated by two considerations: Firstly, the 5 MW turbine size aligns with those commonly found in existing wind farms in the North Seas^15^. Secondly, the choice of a 15 MW turbine is justified by its close resemblance in size to the 14 MW turbine installed in the Haliade-X Dogger Bank. The primary objective of this study is to delve into potential disparities in the near-surface climate impact between employing a low turbine density of larger wind turbines and a high-density configuration of smaller turbines. Additionally, we assess how wake effects affect power generation in scenarios using fewer, larger turbines compared to those with numerous, smaller turbines.

## Experimental design

The regional atmospheric model COSMO-CLM^33^, which includes a wind farm parameterization^14,15,34,35^, was used to investigate the interactions between OWFs and the boundary layer, as well as the wakes generated by them. COSMO-CLM solves the non-hydrostatic compressible primitive equations on an Arakawa-C staggered grid, with a uniform horizontal grid spacing of 0.02˚ (~ 2 km; 396 × 436 grid cells) and a stretched grid spacing in the vertical with 62 terrain following model levels. This allows for 5 and 8 vertical levels within the rotor areas of 5 MW and 15 MW turbines, respectively. Time integration was performed using a third order Runge–Kutta scheme with a time step of 12 s, and vertical turbulent diffusion was parametrized using a one-dimensional prognostic TKE advection scheme^36^. The model used a delta-two-stream scheme for longwave and shortwave radiation with a cloud microphysics scheme^36^. Initial and boundary conditions for the model were taken from the coastDat3 simulations^37^, which are available at hourly intervals on a 0.11˚ grid and are driven by European Centre for Medium-Range Weather Forecast (ECWMF) ERA-Interim reanalysis dataset^38^. Additionally, to the atmospheric forcing the model is forced at the bottom boundary by prescribed sea surface temperature, and the surface roughness over sea is calculated using the Charnock formula^39^.

The wind farm parameterization in COSMO-CLM treats wind turbines as a momentum sink on the mean flow, converting kinetic energy (KE) into electrical energy and TKE ^15,34,35^. The magnitude of the momentum sink varies with wind speed, air density, turbine density, thrust coefficients, and power coefficients. The thrust and power coefficients, which are functions of wind speed, are derived from the National Renewable Energy Laboratory (NREL) 5 MW and International Energy Agency (IEA) 15 MW reference wind turbines for offshore system development^40,41^. Table 1 provides key parameters, such as values of cut-in, cut-out, and rated wind speeds, hub heights, rotor diameters, and turbine density, for both turbines used in this study. A comprehensive validation of the wind speed simulated by COSMO-CLM and the simulated wakes against available observations was conducted in a prior study^15^.

**Table 1:** Key parameters of the turbines used in the experiments.

	5 MW	15 MW
Hub height	90 m	150 m
Rotor diameter	126 m	240 m
Cut-in wind speed	3 ms^–1^	3 ms^–1^
Rated wind speed	11.4 ms^–1^	10.5 ms^–1^
Cut-out wind speed	25 ms^–1^	25 ms^–1^
Turbine density	1.8 km^–2^	0.6 km^–2^
Installed power	108 GW	109 GW

We simulated the atmospheric conditions of 2008–2009 with all existing and planned OWFs in the North Sea for a technical future scenario created in 2015^42^ (see Fig. SI 1). Two simulations were conducted with different wind turbine configurations: 5 MW and 15 MW with turbine densities of 1.8 km^-2^ and 0.6 km^-2^, respectively, resulting in an installed power capacity of 108 GW (Table 1). The COSMO-CLM simulations with wind farm parameterization that includes 15 MW, and 5 MW wind turbine characteristics are referred to as "CCLM_WF15″ and "CCLM_WF5″, respectively. A simulation without wind farm parameterization as control experiment is referred to as "CCLM”.

## Results and discussion

This section provides a detailed discussion of the impact of wakes generated by 15 MW and 5 MW wind turbines on 10 m wind speed, turbulent kinetic energy, 2 m temperature, 2 m specific humidity, and net surface heat flux (NH) and its components at the sea. The North Sea experiences significant spatial and temporal variability in wind speed, with stronger winds in the western regions and during winter. Our simulated wind speeds were validated against observations in a previous study^15^ that extensively investigated wind farms' influence on air-sea heat and momentum fluxes. Here, we focus specifically on changes in air-sea fluxes due to fewer and larger wind turbines^14^. As the focus of the wind industry remains effective energy conversion to electrical power, we add an analysis of the effects of changed wake effects on capacity factors.

**Figure 1:**
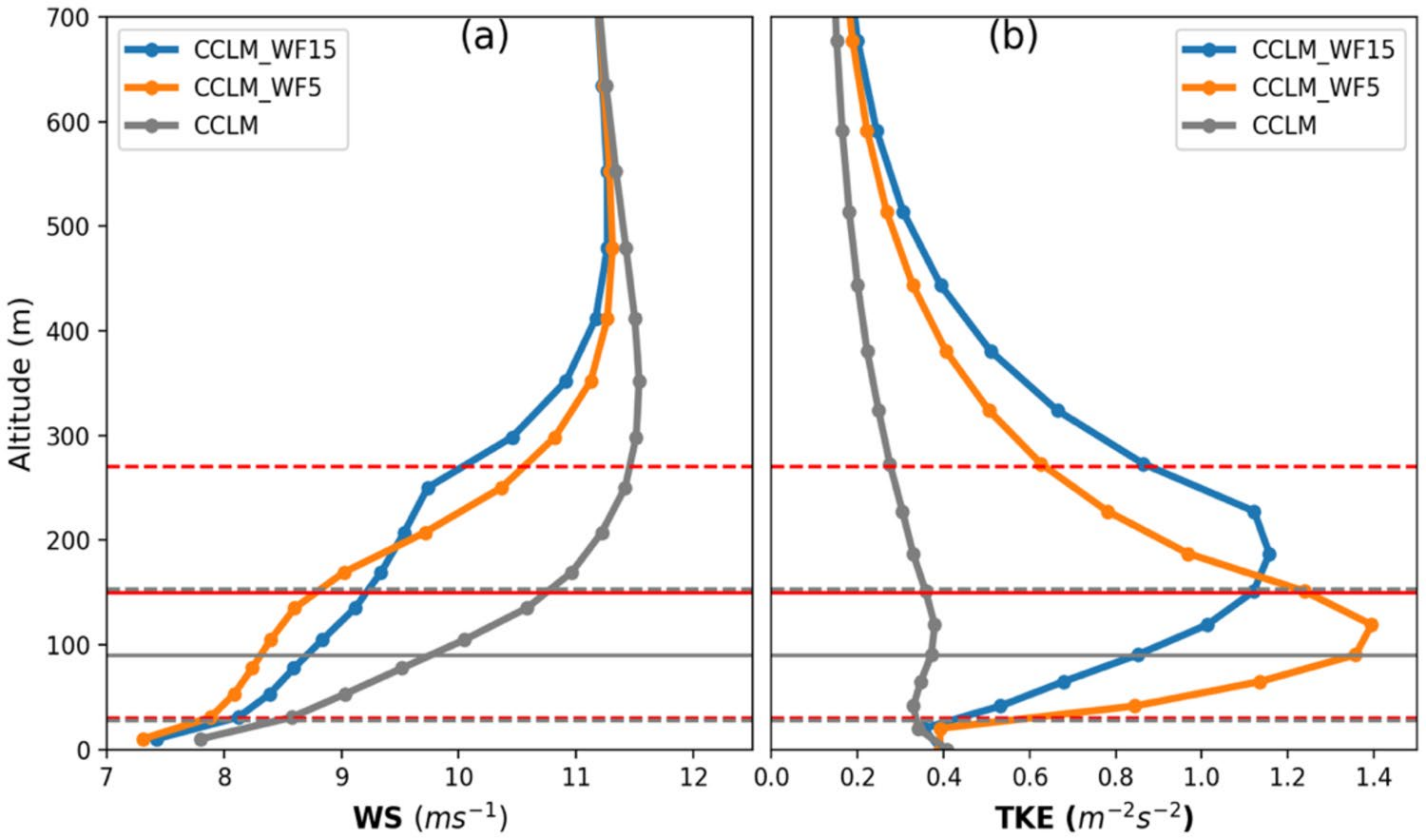
Mean vertical profiles of the CCLM_WF15, CCLM_WF5, CCLM for (**a**) wind speed and (**b**) turbulent kinetic energy over the wind farm areas during the period of 2008–2009. The solid circles indicate the main levels (**a**) or half levels (**b**) of the model. The solid gray line (red lines) indicates the 90 m (150 m) hub height of the turbine, while the dotted gray (red) lines represent the lower tip of the rotor at 27 m (30 m) and upper tip of the rotor at 153 m (270 m) for the 5 MW (15 MW) turbines.

The impact of wind farms on the atmosphere reaches up to 600 m above the sea surface, as shown in Fig. 1. The vertical profiles of mean horizontal wind speed and TKE over the wind farm areas indicate that the highest change in wind speed and TKE occurs between the hub height and the upper tip of the blade. The maximum wind speed reduction due to wake effects is found to be about 14% in CCLM_WF15 and about 18% in CCLM_WF5 over the wind farms. The increase in TKE is about threefold in CCLM_WF15 and fourfold in CCLM_WF5 in the wind farm areas. The increase in TKE and reduction in wind speed is weaker in CCLM_WF15 compared to CCLM_WF5 due to less turbine density.

### Impact of OWFs on 10 m wind speed and TKE

The impact of wind turbines' wakes extends beyond their rotor area and affects the near-surface climate. Recent research has demonstrated that 10 m wind speed is reduced in the wake areas and TKE is slightly reduced at the lowest atmospheric level^14^. Additionally, an acceleration in 10 m wind speed is observed at the upstream edge of wind farms due to the wind channeling effect, which is more pronounced for southwesterly winds. The results indicate that taller and larger wind turbines, combined with their lower density, have a lesser impact on surface wind speed.

**Figure 2:**
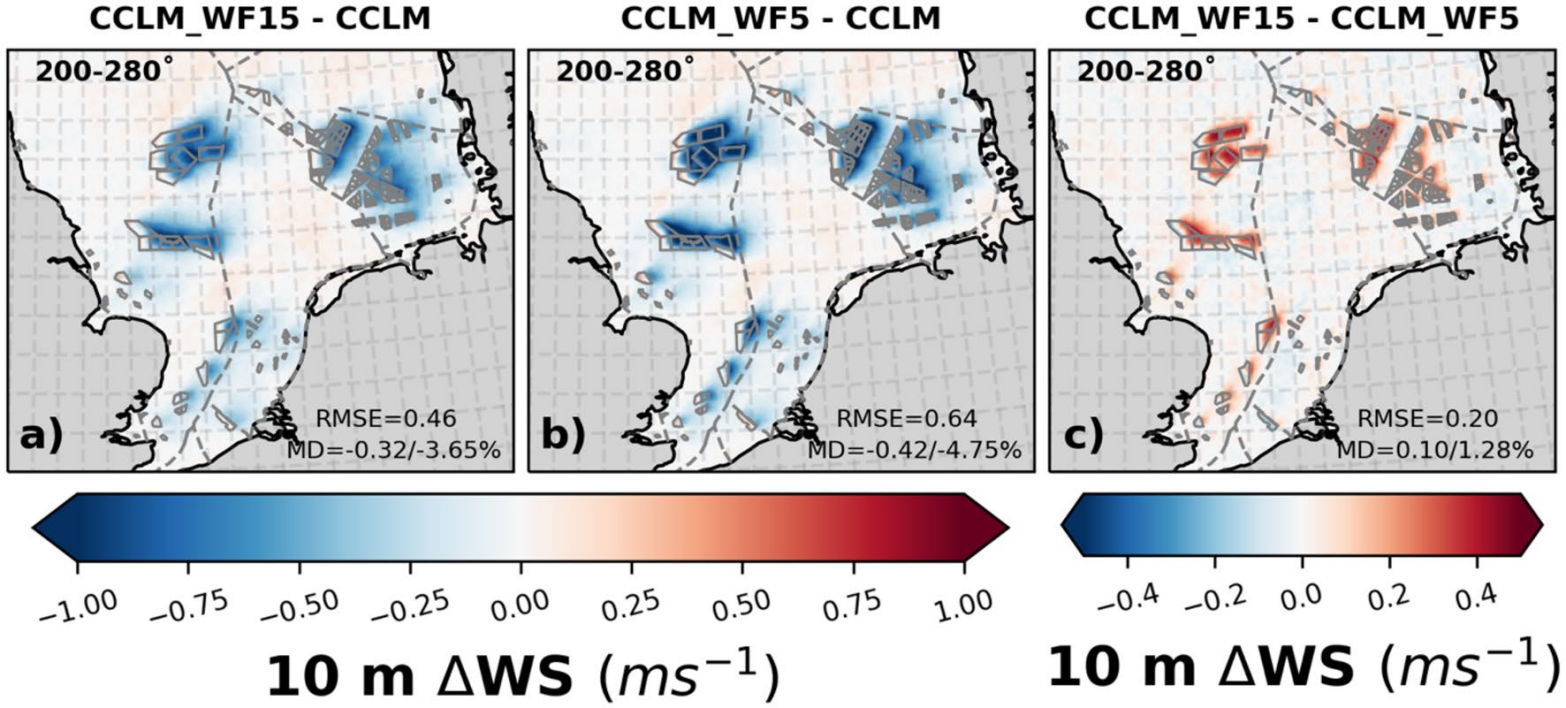
The mean difference of 10 m wind speed between (**a**) CCLM_WF15 and CCLM, (**b**) CCLM_WF5 and CCLM, and (**c**) CCLM_WF15 and CCLM_WF5 for southwesterly winds (200–280°) for the period of 2008–2009. The legend provides root mean square errors (RMSE) and mean differences (MD) over the wind farm areas for the same period. This figure was created using Matplotlib (Hunter, J. D., Matplotlib: a 2D graphics environment. Computing in Science and Engineering 9, 2007) and Cartopy (Met office, Cartopy: a cartographic python library with a matplotlib interface. Exeter, Devon, https://scitools.org.uk/cartopy, 2015).

The comparison between CCLM_WF15 and CCLM_5MW wind farms shows that the reduction in 10 m wind speed is smaller by 0.2–0.4 ms^–1^ (about 1.3%), for wind farms with fewer and larger turbines than for those with many smaller turbines (Fig. 2) in case of southwesterly winds. For all wind directions these differences are about 1.5% (Fig. SI 2). This is due to the lower turbine density, higher hub height, and the greater distance between the lower tip of the blade and the sea surface, which is approximately 3 m more for CCLM_WF15 than for CCLM_5MW wind turbines (Table 1).

The acceleration in wind speed at the upstream edge of the wind farms is slightly weaker for CCLM_WF15 compared to CCLM_WF5. It is worth noting that both observations and model simulations have found near-surface wind speed acceleration^14,43,44^. However, when the wind speed analysis includes all wind directions this near-surface acceleration in wind speed only leads to areas with diminished reduction at the OWF borders, as shown in Fig. SI 2.

**Figure 3:**
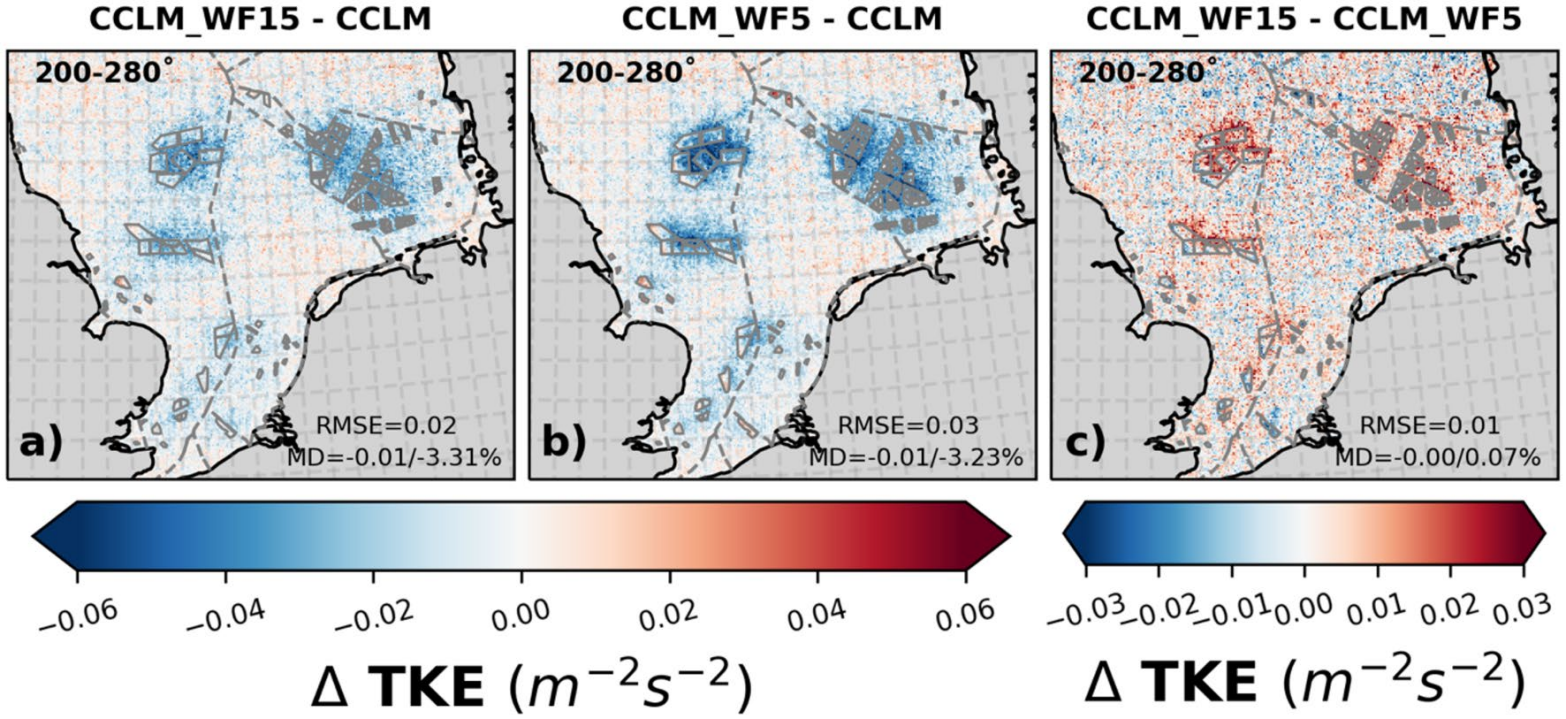
The mean difference of turbulent kinetic energy at lowest atmospheric level between (**a**) CCLM_WF15 and CCLM, (**b**) CCLM_WF5 and CCLM, and (**c**) CCLM_WF15 and CCLM_WF5 for southwesterly winds (200–280°) for the period of 2008–2009. The legend provides root mean square errors (RMSE) and mean differences (MD) over the wind farm areas for the same period. This figure was created using Matplotlib (Hunter, J. D., Matplotlib: a 2D graphics environment. Computing in Science and Engineering 9, 2007) and Cartopy (Met office, Cartopy: a cartographic python library with a matplotlib interface. Exeter, Devon, https://scitools.org.uk/cartopy, 2015).

The conversion of kinetic energy (KE) into electric power by wind turbines results in a reduction of the wind speed and an increase in turbulent kinetic energy (TKE) within the wind farm and downwind wakes^14,34^. The vertical profiles reveal that TKE exhibit different behavior in the lowest layer compared to the layers within the rotor area and at hub height. Figure 3 illustrates the near surface differences in TKE between offshore wind farms and wake areas, compared to boundary-layer flow without wind farms, for southwesterly winds (200–280°). For the wind farm areas CCLM_WF15 and CCLM_WF5 show similar reduced values of surface TKE compared to CCLM of about 3.3% and 3.2% respectively. In the wake areas the reduction of TKE is stronger for the 5 MW turbines. Similar differences are found in case of mean turbulent kinetic energy for all wind directions (Fig. SI 3).

### Impact of OWFs on 2 m specific humidity and temperature

When wind turbines operate, they generate turbulence in the air around them, which can cause the upward movement of moist air^45^. Studies have shown that wind turbines can lead to a decrease in annual mean temperature and an increase in specific humidity above the hub height over the wind farms. This is because the upward movement of moist air can cause it to cool and release its moisture, which can then mix with the drier air from higher altitudes^14^. Wind turbines have been found to alter the vertical profile of the atmosphere primarily within the wind farm area through increased vertical mixing. As a result, the atmospheric levels below the hub height become drier and warmer, with the most significant changes occurring at the edge of the lowest rotor tip.

**Figure 4:**
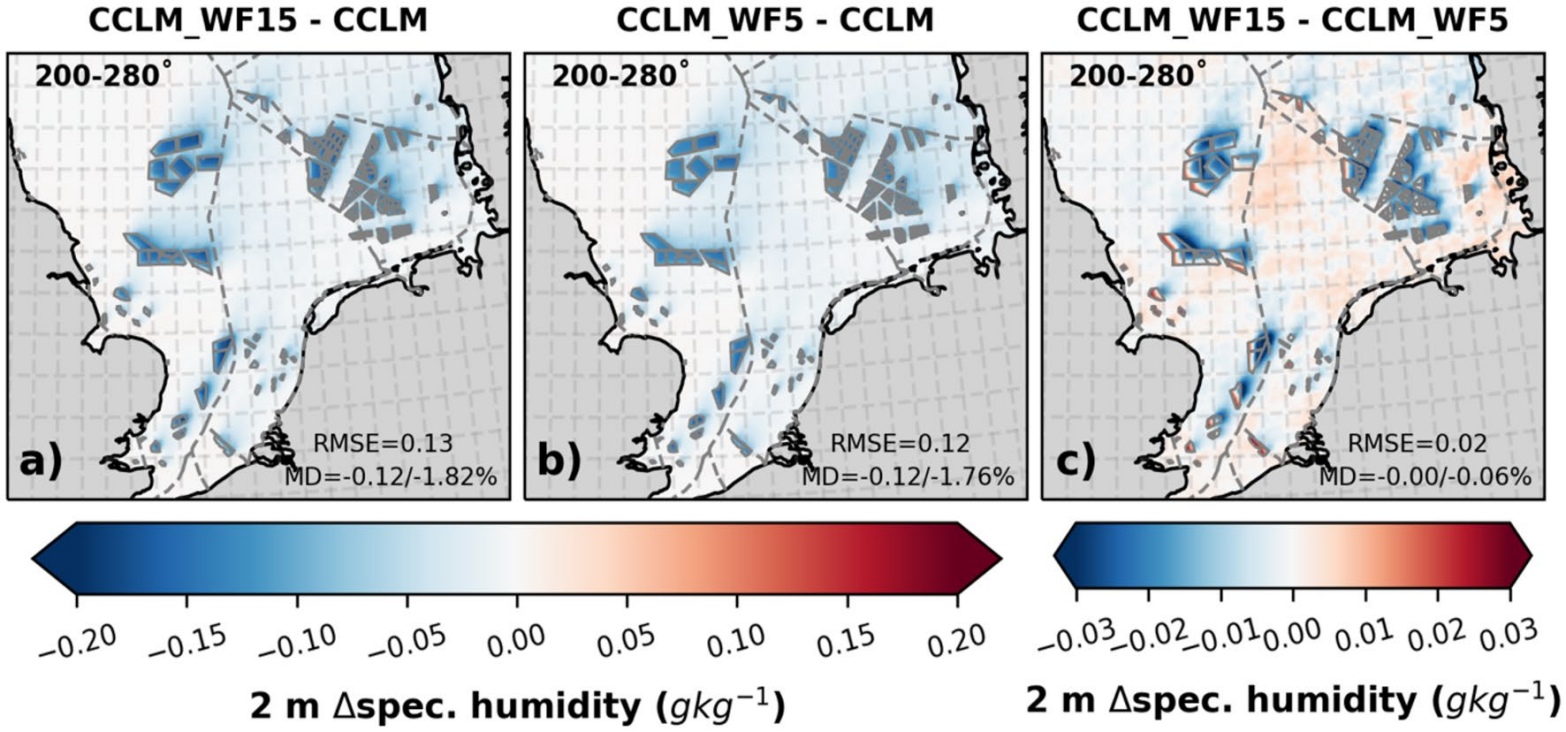
The mean difference of 2 m specific humidity between (**a**) CCLM_WF15 and CCLM, (**b**) CCLM_WF5 and CCLM, and (**c**) CCLM_WF15 and CCLM_WF5 for southwesterly winds (200–280°) for the period of 2008–2009. The legend provides root mean square errors (RMSE) and mean differences (MD) over the wind farm areas for the same period. This figure was created using Matplotlib (Hunter, J. D., Matplotlib: a 2D graphics environment. Computing in Science and Engineering 9, 2007) and Cartopy (Met office, Cartopy: a cartographic python library with a matplotlib interface. Exeter, Devon, https://scitools.org.uk/cartopy, 2015).

In case of southwesterly winds, the annual mean difference in specific humidity at 2 m was found to be reduced by up to 0.12 g kg^−1^ (1.8%) in the wind farm areas and wakes of the CCLM_WF15 simulation compared to the CCLM simulation, as shown in Fig. 4. Furthermore, compared to the CCLM_WF5 simulation, the differences in 2 m specific humidity were slightly lower (ranging from 0.02 to 0.03 g kg^–1^) in the downwind stream and slightly higher at the upstream edges of the wind farm in the CCLM_WF15 simulation. Including all wind directions, the differences in 2 m specific humidity between the CCLM_WF15 and CCLM_WF5 simulations are very small, approximately 0.01 g kg^–1^ or 0.1% as shown in Figure SI 4.

**Figure 5:**
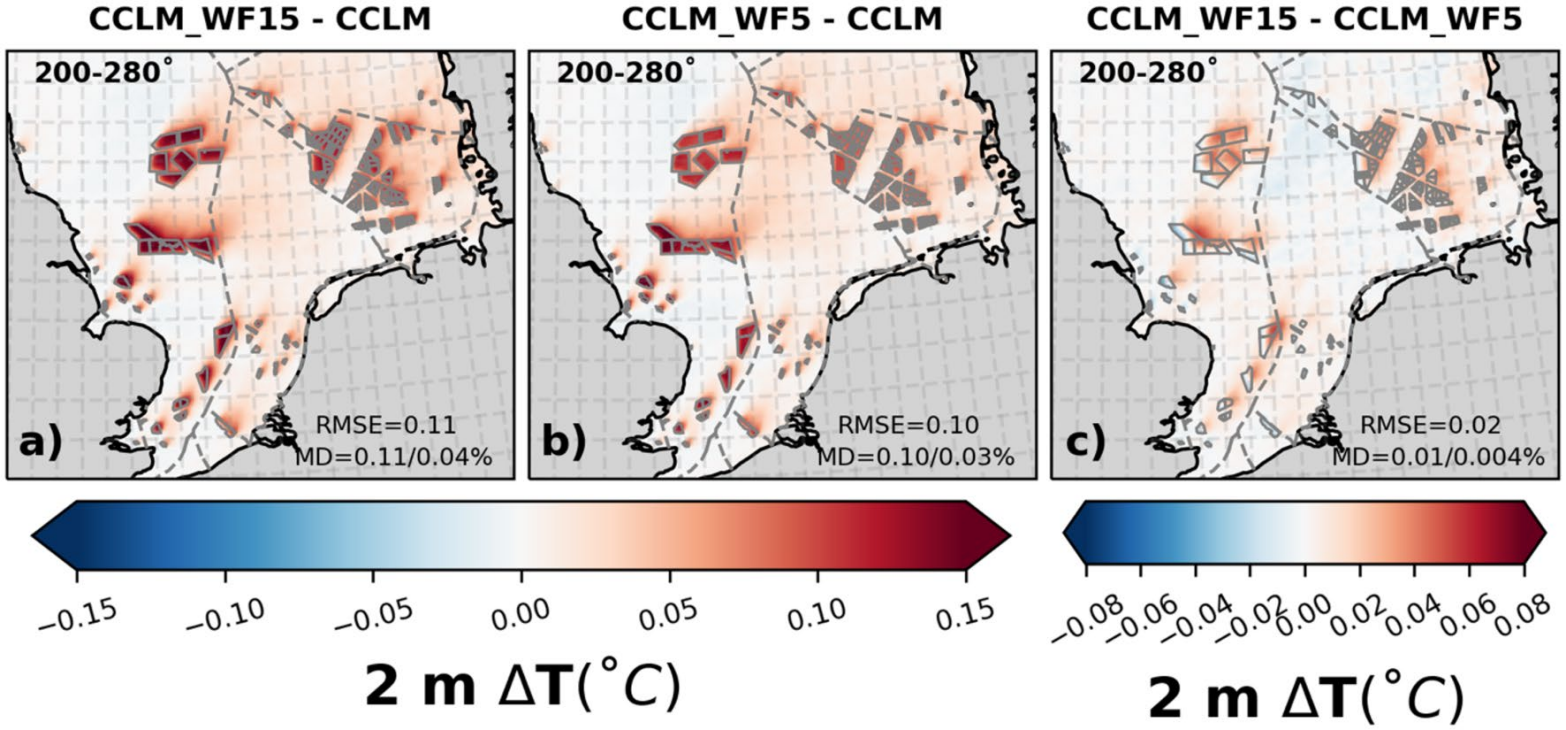
The mean difference of 2 m temperature between (**a**) CCLM_WF15 and CCLM, (**b**) CCLM_WF5 and CCLM, and (**c**) CCLM_WF15 and CCLM_WF5 for southwesterly winds (200–280°) for the period of 2008–2009. The legend provides root mean square errors (RMSE) and mean differences (MD) over the wind farm areas for the same period. This figure was created using Matplotlib (Hunter, J. D., Matplotlib: a 2D graphics environment. Computing in Science and Engineering 9, 2007) and Cartopy (Met office, Cartopy: a cartographic python library with a matplotlib interface. Exeter, Devon, https://scitools.org.uk/cartopy, 2015).

The increase in 2 m temperature observed in the wind farm areas (Fig. 5) is caused by the decrease in moisture resulting from enhanced vertical mixing. Specifically, the annual mean temperatures at 2 m show an increase of up to 0.11 °C primarily in the wind farm areas in the CCLM_WF15 simulation compared to the CCLM simulation. The differences in 2 m temperature between the CCLM_WF15 and CCLM_WF5 simulations are small, about 0.01 °C for the prevailing winds as for all wind directions (Fig. SI 5).

### Impact of OWFs on net surface heat fluxes

The impact of wind turbines on near-surface wind speed and TKE affects the net heat flux in the atmosphere by modifying the turbulent fluxes such as the latent heat (LH) and the sensible heat (SH) fluxes, as well as radiative fluxes including shortwave (SW) and longwave (LW) radiation^14^. The changes in these fluxes are mainly influenced by the change in 10 m wind speed, TKE, specific humidity, and temperature gradient between the surface and the lowest atmospheric level. The decrease in TKE results in a reduction in mixing in the lowest atmospheric layer, which leads to a decrease in turbulent fluxes^14^.

**Figure 6:**
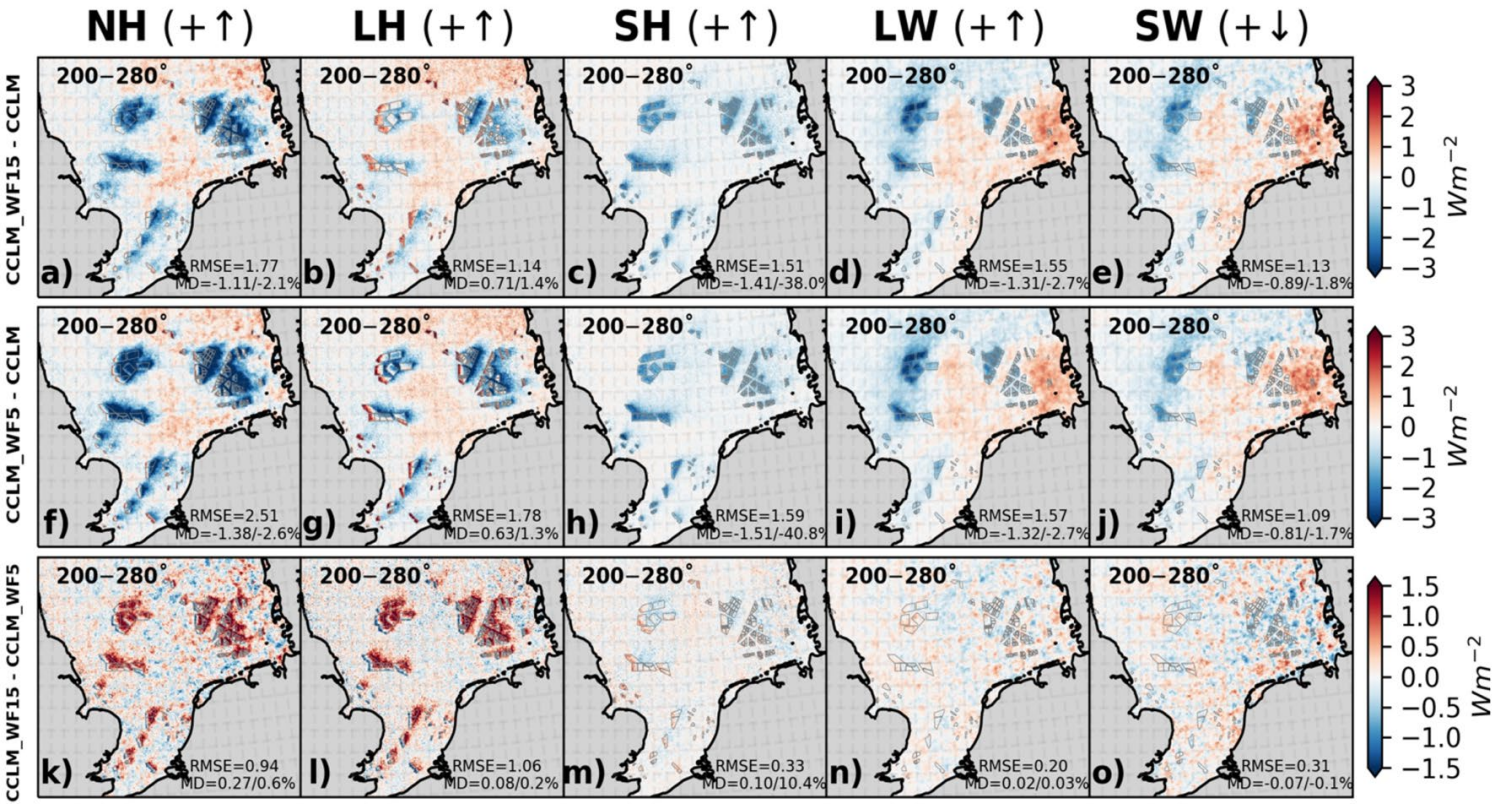
Mean differences between CCLM_WF15 and CCLM (first row), CCLM_WF5 and CCLM (second row), and CCLM_WF15 and CCLM_WF5 (third row) for (fist column) net heat (NH) flux, (second column) latent heat (LH) flux, (third column) sensible heat (SH) flux, (fourth column) net upwelling longwave (LW) radiation, and (fifth column) net shortwave downwelling (SW) radiation for wind directions of 200–280° during the period of 2008–2009. The legend provides root mean square errors (RMSE) and mean differences (MD) over the wind farm areas for the same period. This figure was created using Matplotlib (Hunter, J. D., Matplotlib: a 2D graphics environment. Computing in Science and Engineering 9, 2007) and Cartopy (Met office, Cartopy: a cartographic python library with a matplotlib interface. Exeter, Devon, https://scitools.org.uk/cartopy, 2015).

The comparison between CCLM_WF15 and CCLM indicates an average increase of LH flux by about 0.7 Wm^-2^ (1.4%) in the wind farm areas during southwesterly winds (Fig. 6). However, locally, these differences vary between − 1.5 and 1.5 Wm^–2^. Similarly, when comparing CCLM_WF5 and CCLM, the differences are around 0.6 Wm^−2^ (1.3%), varying from − 3 to 3 Wm^–2^. The increase in LH flux mainly occurs in areas where the upstream wind speed accelerates. Figure 6 (third row) shows that larger and fewer wind turbines cause a slightly stronger increase in LH flux, with only a 0.2% difference compared to smaller turbines. For winds from all directions, the LH flux difference between CCLM_WF15 and the standard CCLM version is increased by 0.6% (Fig. SI 6).

According to the results, the SH flux in CCLM_WF15 causes a reduction of approximately 1.4 Wm^–2^ (38%) when compared to CCLM for southwesterly winds (Fig. 6). The difference between CCLM_WF5 and CCLM is approximately − 1.5 Wm^–2^ (40.8%). This suggests that the reduction in SH is slightly less in CCLM_WF15, at around 0.10 Wm^–2^ (10.4%), compared to CCLM_WF. When considering the mean winds in all directions, the reduction in SH flux in CCLM_WF15 is about 0.11 Wm^–2^ (2.5%) weaker when compared to CCLM_WF5 (Fig. SI 6).

The installation of fewer and larger wind farms also affects the SH flux by causing less reduction in 10 m wind speed (Fig. 2) and changes in 2 m temperature (Fig. 5). In comparison to CCLM_WF5, the reduction in SH is lower in CCLM_WF15 by approximately 0.1 Wm^–2^ (10.4%) for southwesterly winds (Fig. 6) and about 0.11 Wm^–2^ (2.5%) in mean for all wind directions (and SI 6). The SH flux is primarily influenced by the temperature gradient between the sea surface and the lowest atmospheric layer, in addition to wind speed, as noted in previous studies^14^.

Changes in the specific humidity/temperature contrast between the surface and the lowest atmospheric layer are the main cause of changes in the surface turbulent fluxes (Fig. 6). Variations in wind speed also indirectly impact these fluxes. Furthermore, the decrease in TKE in the lowest atmospheric layer (Fig. 3) results in a reduction of the turbulent diffusion coefficient for heat.

Studies employing the same wind farm parameterization utilized in this study have similarly documented a reduction in the LH flux when the lowest atmospheric layer is warmer and drier compared to the surface layer, particularly for onshore wind farms^45,46^. Likewise, a numerical study conducted using the mesoscale model METRAS over the German Bight region identified a decrease in turbulent fluxes^47^. Additionally, experimental evidence derived from wind-tunnel experiments substantiates a reduction in surface heat fluxes by approximately 4%, in the context of staggered wind farms^48^. Conversely, when the lower atmospheric layer is moister and colder than the surface layer, an increase in LH heat fluxes has been observed^49^.

Wind farms can indeed exert an influence on low-level clouds, subsequently affecting radiative fluxes^14^. Low clouds tend to increase because of flow convergence and uplift occurring at the upstream edge of wind farms^45^. The turbulence generated by wind turbines results in increased moisture at higher altitudes, leading to heightened relative humidity and cloud coverage due to adiabatic cooling. An increase in low-level clouds above the wind farms is also evident in our findings (Fig. SI 7). The annual mean values differ slightly for the WF simulations and show an increase of up to approximately 4.2% in low clouds in the CCLM_WF15 and in CCLM_WF5 compared to the CCLM over the wind farm areas for southwesterly winds. For all wind directions, the difference between CCLM_WF15 and CCLM_WF5 is low as well with approximately 0.1%. This additional formation of clouds above wind farms has also been observed^50^.

The primary effect of an increase in clouds is to reduce incoming solar radiation (Figs. 6 and SI 6). There's a minimal disparity in the impact on radiative fluxes between CCLM_WF15 and CCLM_WF5, particularly when considering southwesterly winds (Fig. 6). When examining the overall averages across varying wind conditions, we observe only slight deviations in their influence on radiative fluxes due to the presence of fewer but larger wind turbines, as depicted in Figure SI 6. Furthermore, cloud cover and precipitation exhibit similar patterns in their mean values when subjected to winds from all directions, as revealed in Figures SI 8 and SI 9. The alteration in cloud patterns, characterized by an increase over the wind farms and a subsequent reduction downwind of the wind farms, has been reported in previous studies^47^.

### Impact of OWFs on capacity factor

The increase in wind speed with altitude from 90 to 150 m height might lead to an increase in the CF of power production by about 3–5% (Fig. SI 10) without wake effects. This is because the wind is stronger and more stable in greater heights, which gives the possibility for optimum power generation by the installation of taller and larger wind turbines, such as 15 MW turbines with a 150 m rotor height and 240 m diameter. However, it is also important to estimate the impact of wakes generated by these wind turbines on power generation.

**Figure 7:**
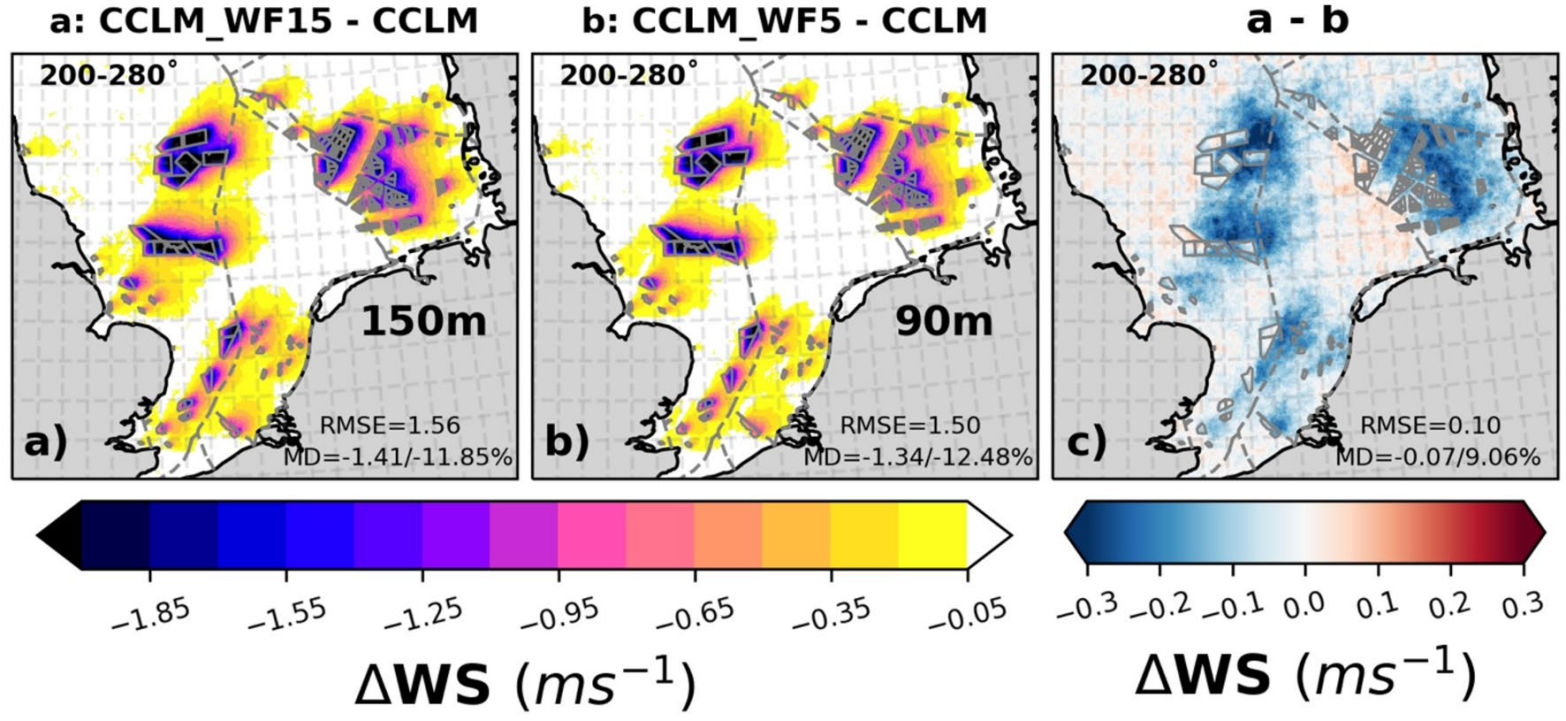
The mean difference of wind speeds at hub height between (**a**) CCLM_WF15 (150 m) and CCLM, (**b**) CCLM_WF5 (90 m) and CCLM, and (**c**) CCLM_WF15 and CCLM_WF5 for southwesterly winds (200–280°) during the period of 2008–2009. The legend provides root mean square errors (RMSE) and mean differences (MD) over the wind farm areas for the same period. This figure was created using Matplotlib (Hunter, J. D., Matplotlib: a 2D graphics environment. Computing in Science and Engineering 9, 2007) and Cartopy (Met office, Cartopy: a cartographic python library with a matplotlib interface. Exeter, Devon, https://scitools.org.uk/cartopy, 2015).

Figure 7 shows that the reduction in the annual mean wind speed is notable, reaching up to 2–2.5 ms^–1^ during prevailing southwesterly winds (200°–280°). The mean differences between CCLM_WF15 and CCLM are approximately − 1.4 ms^–1^ (11.8%), and between CCLM_WF5 and CCLM, it is about − 1.3 ms^–1^ (12.5%). The wakes generated in CCLM_WF15 are slightly stronger (0.07/9.0%) in magnitude and larger in extent than the wakes generated in CCLM_WF5 at their respective hub heights of 150 and 90 m for prevailing southwesterly wind directions. On average, wakes extend approximately 40–45 km downwind^15^. Observational evidence indicates that, depending on wind speed and atmospheric conditions, the wake may extend more than 70 km downwind^22,30^. Despite the turbine density in CCLM_WF15 being about three times lower than in CCLM_WF5, the wakes generated by the wind farms installed with 15 MW turbines are stronger by about 0.2–0.4 ms^–1^ downwind of the wind farms and more far reaching due to the larger rotor diameter (Table 1). Over the wind farm area, the differences in wakes generated by CCLM_WF15 and CCLM_WF5 are minor. The differences in wake strength with regard to wind speed between the two wind farm configurations is around 4% over the wind farms areas when considering all wind conditions (Fig. SI 11).

**Figure 8:**
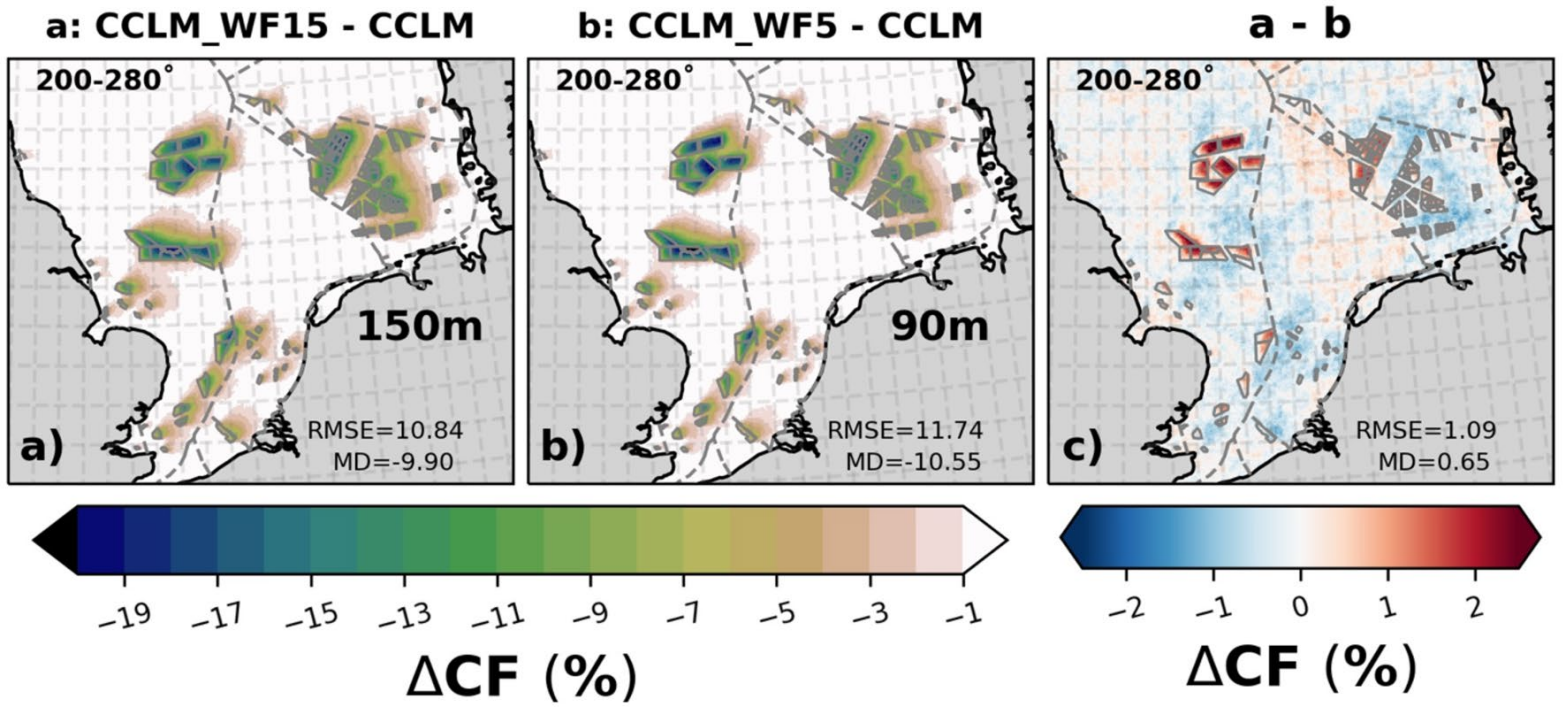
The mean differences of capacity factor at hub height between (**a**) CCLM_WF15 and CCLM, (**b**) CCLM_WF5 and CCLM, and (**c**) CCLM_WF15 and CCLM_WF5 for southwesterly winds (200–280°) during the period of 2008–2009. The legend provides root mean square errors (RMSE) and mean differences (MD) over the wind farm areas for the same period. This figure was created using Matplotlib (Hunter, J. D., Matplotlib: a 2D graphics environment. Computing in Science and Engineering 9, 2007) and Cartopy (Met office, Cartopy: a cartographic python library with a matplotlib interface. Exeter, Devon, https://scitools.org.uk/cartopy, 2015).

Due to the stronger wake effect generated by the wind farms with 15 MW turbines, there is a further reduction in CF by up to 1% downstream of the wind farms (as shown in Fig. 8). However, within the wind farm areas, the CF of CCLM_WF15 is higher (1–2%) than CCLM_WF5 due to a lesser density and better wind conditions at the higher level (not shown). Additionally, the difference in CF within the wind farm is due to the difference in the rated wind speed of the wind turbines, as specified in Table 1. When considering the winds of all directions, CCLM_WF15 shows a slight decrease in CF of about 0.5% outside the wind farm areas compared to CCLM_WF5 (Fig. SI 12). However, inside the wind farm areas, CCLM_WF15 shows an increase of about 1% compared to CCLM_WF5. Considering the 3.5–4% increase in CF from 90 to 150 m there is an overall gain of 4–5% in CF.

The magnitude of wake effects produced by wind farms can exert a substantial influence on adjacent downstream wind farms, especially when the wind farm is situated to the east of them. In cases where windfarms are located near the boundary of an exclusive economic zone (EEZ), the wakes generated by a wind farm can affect the wind resources of the downwind farms located in the EEZ of another country. Therefore, political agreements must be reached to protect the rights of the parties involved.

**Figure 9:**
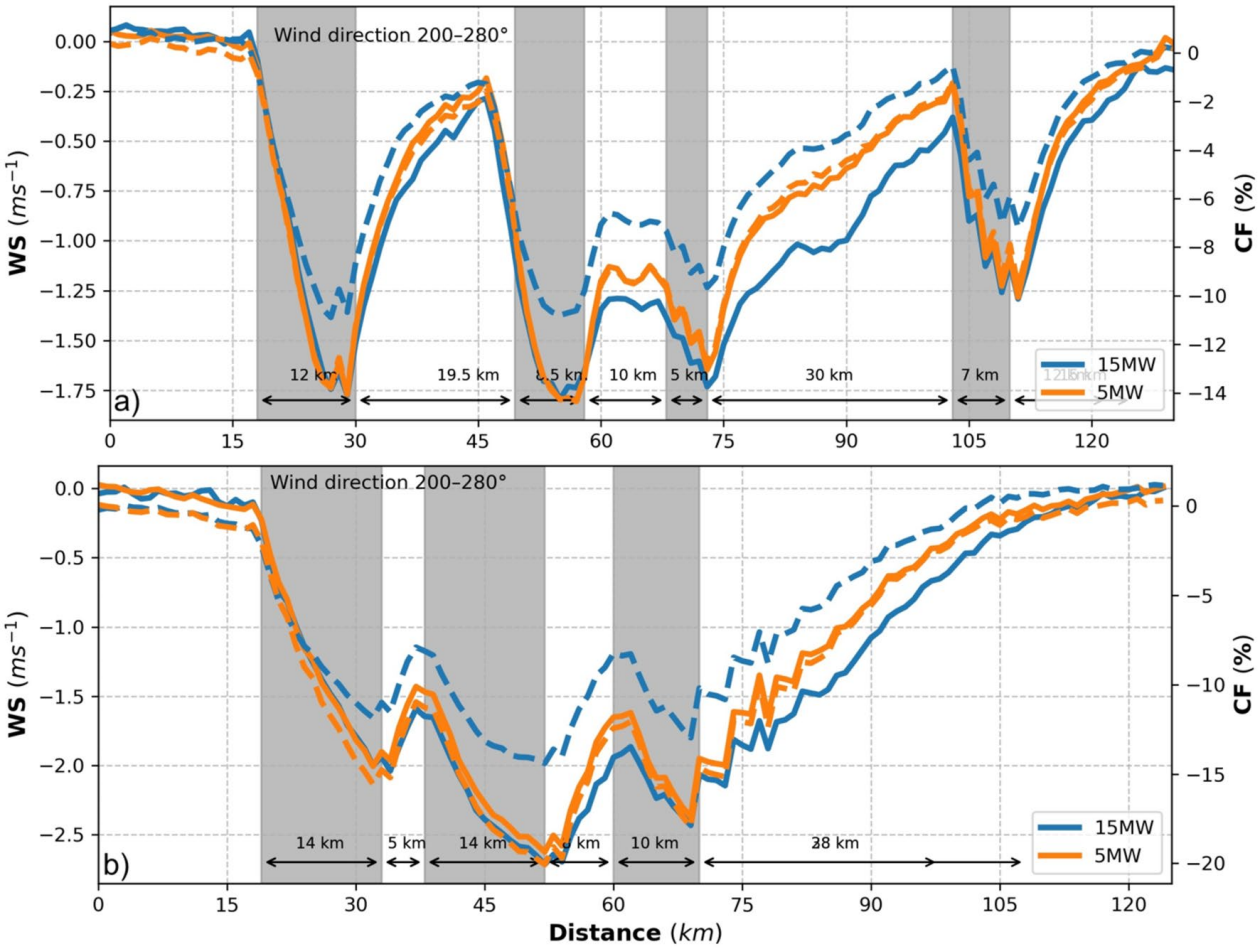
Transects of wind speed (left axis), and CF (right axis) deviations from means at hub height for CCLM_WF15 (blue), and CCLM_WF (orange) for the prevailing wind directions of 200–280° in 2008–2009 taken at (**a**) transect II (see Fig. SI 1) latitude 54.8° N–54.4° N and longitude 4.4° E–8.4° E, and (**b**) transect IV (see Fig. SI 1) latitude 54.8° N–55.35° N and longitude 1.0˚ E–4.10˚ E. Gray sectors indicate the wind farm positions.

Transects analysis of the wind speed deficits and CF at hub height through the wind farms for prevailing southwesterly wind directions show that wind speed deficit can reach up to 35–40 km downwind of the wind farms (Fig. 9). Inside the wind farms, wind speed deficits are smaller in CCLM_WF15 than CCLM_WF5. This results in about 3–4% higher CF in CCLM_WF15. Transects along paths 1 and 3 are shown in the supplementary information (Fig. SI 13).

## Discussion and conclusion

The study compared the wakes generated by wind farms with 15 MW and 5 MW turbines in the North Sea and analyzed the potential to reduce human impact on the near-surface climate and effects on power production. The installed capacity for both scenarios was approximately 108 GW, but the turbine density differed significantly, with the 5 MW scenario having almost three times as many turbines. The rotor diameter of the 15 MW turbines was nearly double that of the 5 MW turbines. The findings suggest that wind farms with fewer and larger turbines increase the power production capacity. However, the impact on near-surface winds and heat flux is slightly less with fewer and larger wind turbines (15 MW) compared to many smaller wind turbines.

The increase in wind speed with changed hub height from 90 to 150 m can lead to a 3–5% increase in the capacity factor of power production, but the wakes generated by the larger turbines result in a reduction in the CF (0.5–1%) downstream of the wind farms. The extent of the wakes generated can have significant impacts on downstream wind farms. The study found that the wakes generated by offshore wind farms with 15 MW turbines are slightly stronger downwind than those generated by 5 MW turbines, with a difference of up to 0.4 ms^–1^. The wakes can extend up to 35–40 km downwind with a maximum wind speed deficit of up to 2–2.5 ms^–1^.

Wind turbines' wakes have a significant impact on the near-surface climate beyond their rotor area. The reduction in 10 m wind speed is smaller for wind farms with fewer and larger turbines than for those with many smaller turbines. This reduced impact on 10 m wind speed (0.2–0.4 ms^–1^) is due to their lower density and higher hub height. It also influences the turbulence fluxes, mainly the latent heat flux. Larger and taller wind turbines have a reduced impact on latent heat flux due to changes in wind speed and turbulent kinetic energy. The impact on sensible heat flux is minimal, and the difference in radiative fluxes between larger and smaller turbines as well. Wind farms can modify low-level clouds, but the impact on cloud fraction and precipitation are similar for 5 MW and 15 MW turbines.

The study suggests that wind farms with larger and taller wind turbines (15 MW) have a reduced impact on near-surface wind speed and heat fluxes compared to wind farms with many smaller wind turbines (5 MW). This could mean that larger wind turbines have less impact on the ocean dynamics and ecosystem, as sea surface winds and heat fluxes are important drivers of these systems. A recent study^31^, employing consistent atmospheric forcing and wind farm scenarios, along with the identical 5 MW turbines used in this experiment, emphasizes the substantial impact of wind wakes on the ecosystem of the North Sea. Ocean and ecosystem modeling studies, employing the strategy of incorporating atmospheric forcing to account for wake effects, aim to enhance our understanding of the potential impacts of various types of wind farms on ocean dynamics and ecosystems.

Furthermore, essential technical advancements are needed to integrate various types of wind turbines into the COSMO-CLM model to achieve more realistic scenarios in wind farm simulations. Moreover, a greater availability of observational data is required for result validation. Additionally, wind farm parametrization in RCMs has inherent limitations, including a simplified representation of wind farms, limited consideration of wake interactions between turbines, coarse spatial resolution, and a lack of feedback between the ocean and the atmosphere.

### Supplementary Information

Supplementary Figures.

## Data Availability

The COSMO-CLM_WF and COSMO-CLM model datasets supporting the results and the COSMO-CLM name lists are available from the authors upon request. The COSMO-CLM simulations employ the community-wide, publicly available (http://www.clm-community.eu) COSMO-CLM code. All the data will be available from corresponding author upon reasonable request.
