# Supplementary material for: Larger wind turbines as a solution to reduce environmental impacts
Source: Sci Rep. 2024 Mar 19;14:6608. doi: 10.1038/s41598-024-56731-w (PMC10950853; doi:10.1038/s41598-024-56731-w)
Supplement: Supplementary file 1 — Supplementary Figures. [file 41598_2024_56731_MOESM1_ESM.docx]

# **Supplementary information**

# **Larger wind turbines as a solution to reduce environmental impact**

Naveed Akhtar^1^*, Beate Geyer^1^, Corinna Schrum^1,2^

^1^ Institute of Coastal Systems - Analysis and Modeling, Helmholtz-Zentrum Hereon, Geesthacht, Germany

^2^ Center for Earth System Research and Sustainability, Institute of Oceanography, University of Hamburg, Germany

*Corresponding author: Naveed Akhtar ([naveed.akhtar@hereon.de](mailto:naveed.akhtar@hereon.de))

Fig. SI 1. The figure shows the configuration of offshore wind farms (OWFs) used in the model scenarios for the North Sea, with colored polygons indicating the planning status of the OWFs by 2015. The land-sea mask of the model domain is also shown. Gray lines indicate the transects used for analysis. The figure was created using the Matplotlib (Hunter, J. D., Matplotlib: a 2D graphics environment. Computing in Science and Engineering 9, 2007) and Cartopy (Met office, Cartopy: a cartographic python library with a matplotlib interface. Exeter, Devon, https://scitools.org.uk/cartopy, 2015) libraries.


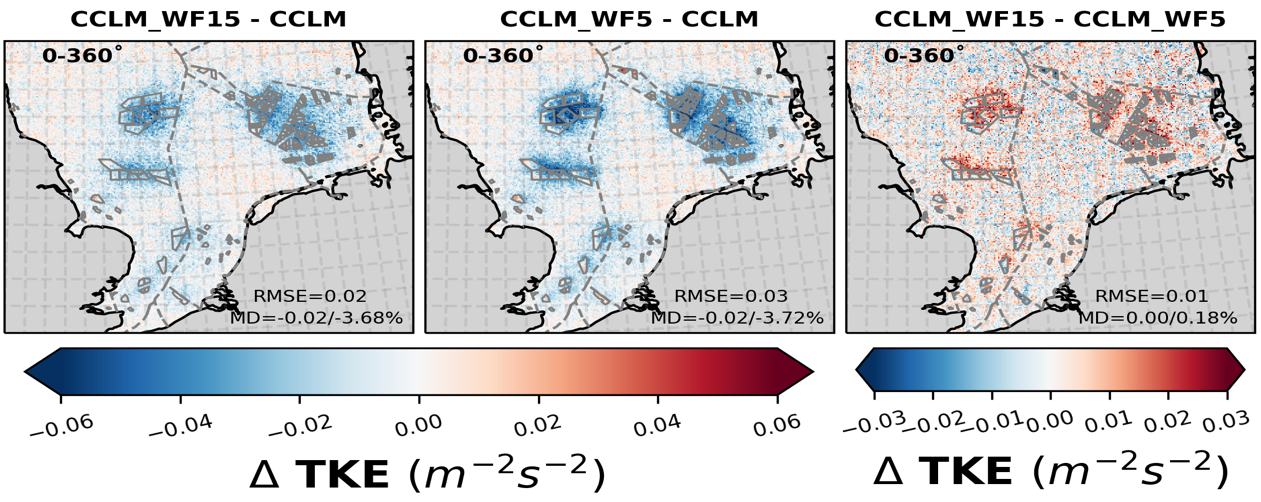

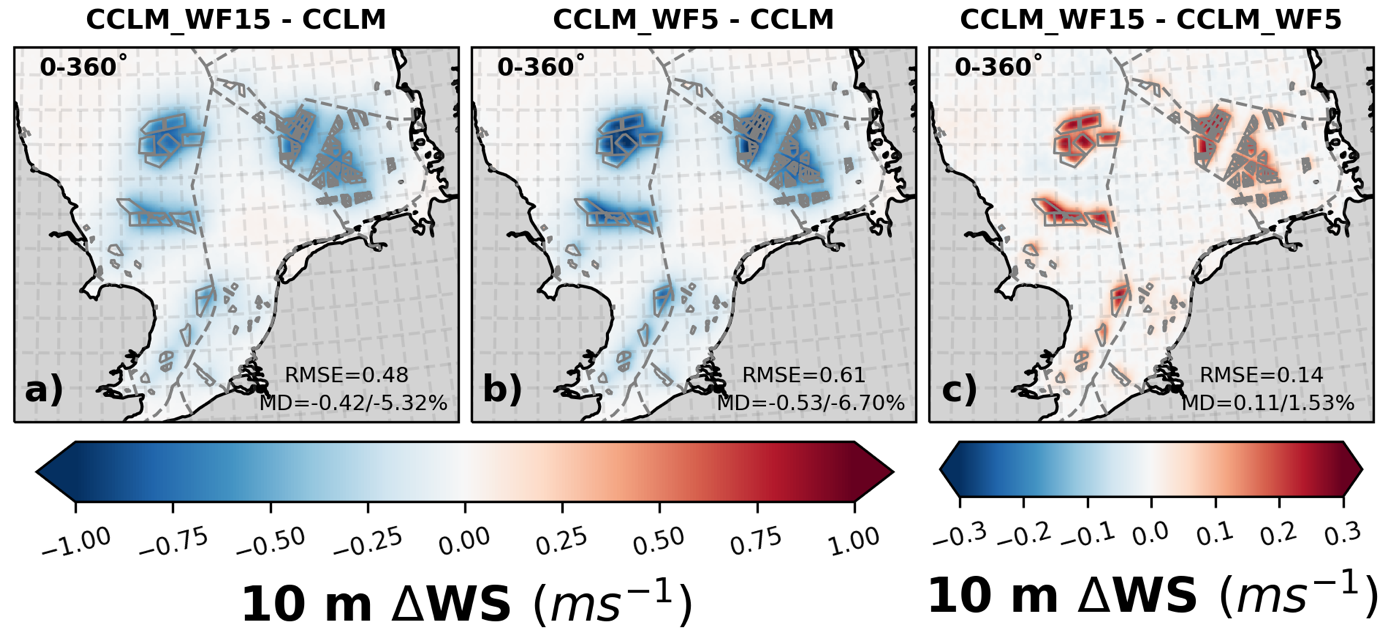


Fig.SI 3. The mean difference of turbulent kinetic energy (TKE) at lowest atmospheric level between a) CCLM_WF15 and CCLM, b) CCLM_WF5 and CCLM, and c) CCLM_WF15 and CCLM_WF5 for all wind directions (0—360˚) during the period of 2008—2009. The legend provides Root Mean Square Errors (RMSE) and Mean Differences (MD) over the wind farm areas for the same period. This figure was created using Matplotlib (Hunter, J. D., Matplotlib: a 2D graphics environment. Computing in Science and Engineering 9, 2007) and Cartopy (Met office, Cartopy: a cartographic python library with a matplotlib interface. Exeter, Devon, https://scitools.org.uk/cartopy, 2015).

Fig. SI 2. The mean difference of 10 m wind speed (WS) between a) CCLM_WF15 and CCLM, b) CCLM_WF5 and CCLM, and c) CCLM_WF15 and CCLM_WF5 for all wind directions (0—360˚) during the period of 2008—2009. The legend provides Root Mean Square Errors (RMSE) and Mean Differences (MD) over the wind farm areas for the same period. This figure was created using Matplotlib (Hunter, J. D., Matplotlib: a 2D graphics environment. Computing in Science and Engineering 9, 2007) and Cartopy (Met office, Cartopy: a cartographic python library with a matplotlib interface. Exeter, Devon, https://scitools.org.uk/cartopy, 2015).


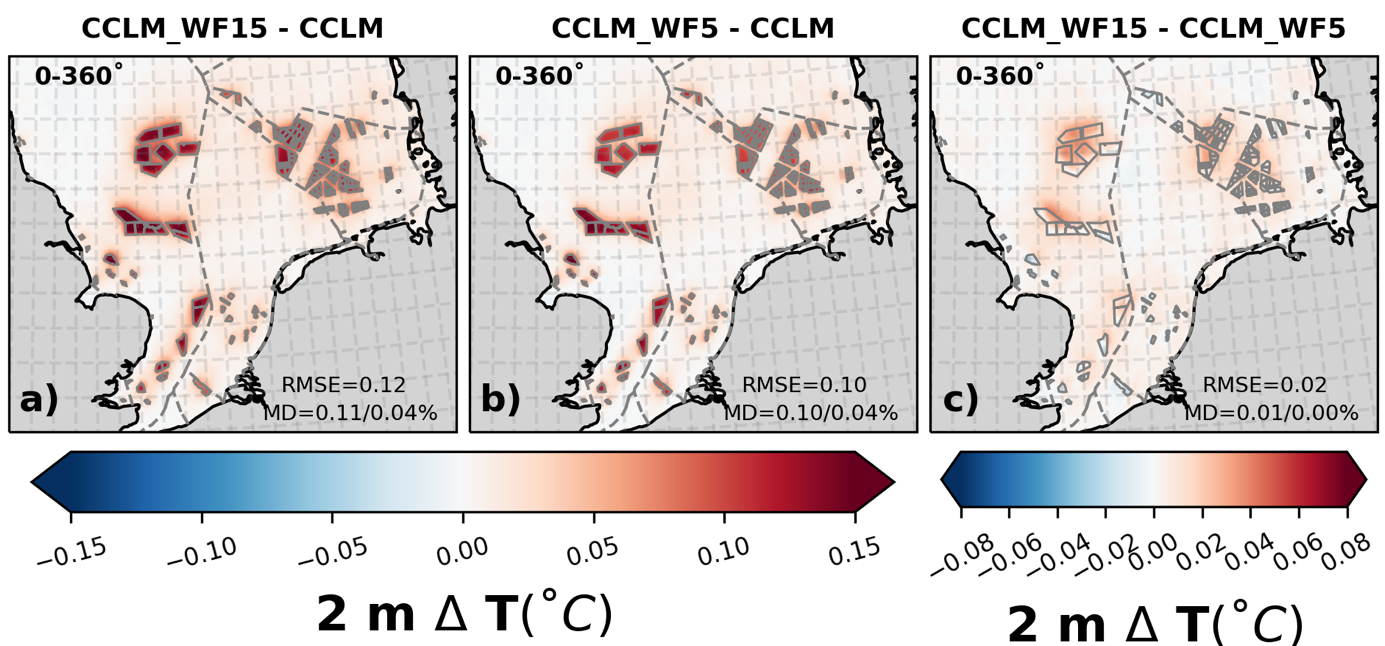

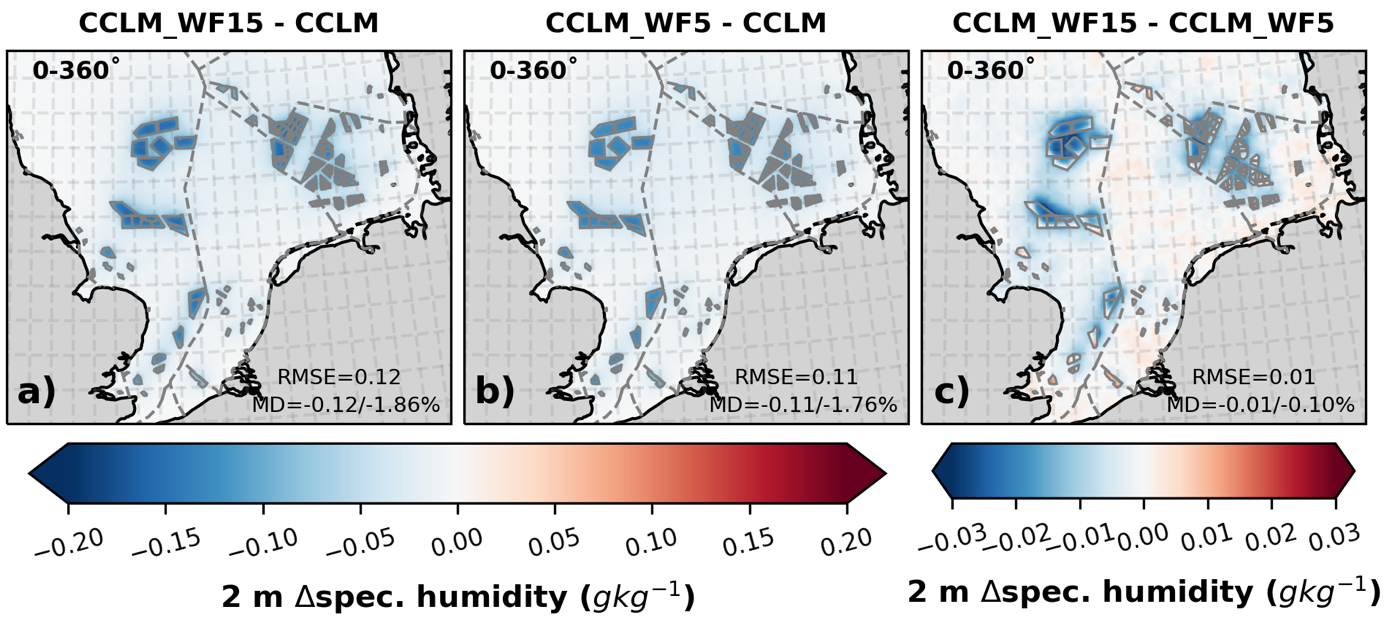


Fig. SI 4. The mean difference of 2 m specific humidity between a) CCLM_WF15 and CCLM, b) CCLM_WF5 and CCLM, and c) CCLM_WF15 and CCLM_WF5 for all wind directions (0—360˚) during the period of 2008—2009. The legend provides Root Mean Square Errors (RMSE) and Mean Differences (MD) over the wind farm areas for the same period. This figure was created using Matplotlib (Hunter, J. D., Matplotlib: a 2D graphics environment. Computing in Science and Engineering 9, 2007) and Cartopy (Met office, Cartopy: a cartographic python library with a matplotlib interface. Exeter, Devon, https://scitools.org.uk/cartopy, 2015).

Fig. SI 5. The mean difference of 2 m temperature (T) between a) CCLM_WF15 and CCLM, b) CCLM_WF5 and CCLM, and c) CCLM_WF15 and CCLM_WF5 for southwesterly winds (0—360˚) during the period of 2008—2009. The legend provides Root Mean Square Errors (RMSE) and Mean Differences (MD) over the wind farm areas for the same period. This figure was created using Matplotlib (Hunter, J. D., Matplotlib: a 2D graphics environment. Computing in Science and Engineering 9, 2007) and Cartopy (Met office, Cartopy: a cartographic python library with a matplotlib interface. Exeter, Devon, https://scitools.org.uk/cartopy, 2015).


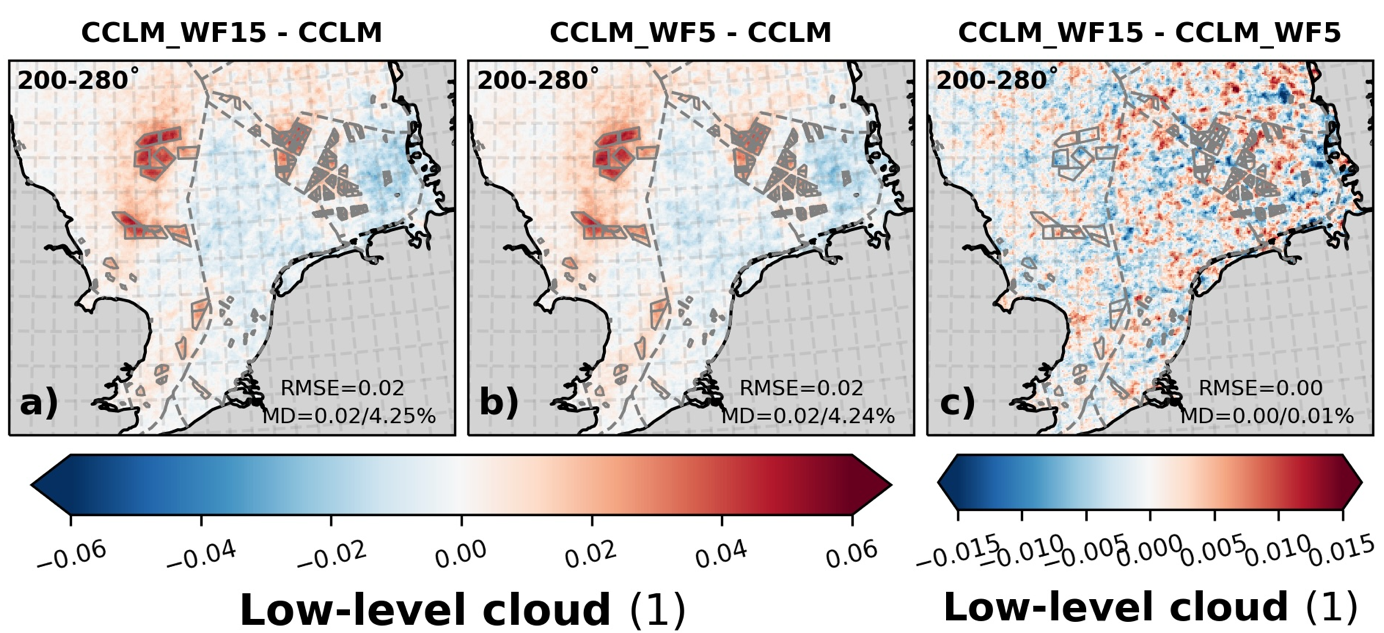

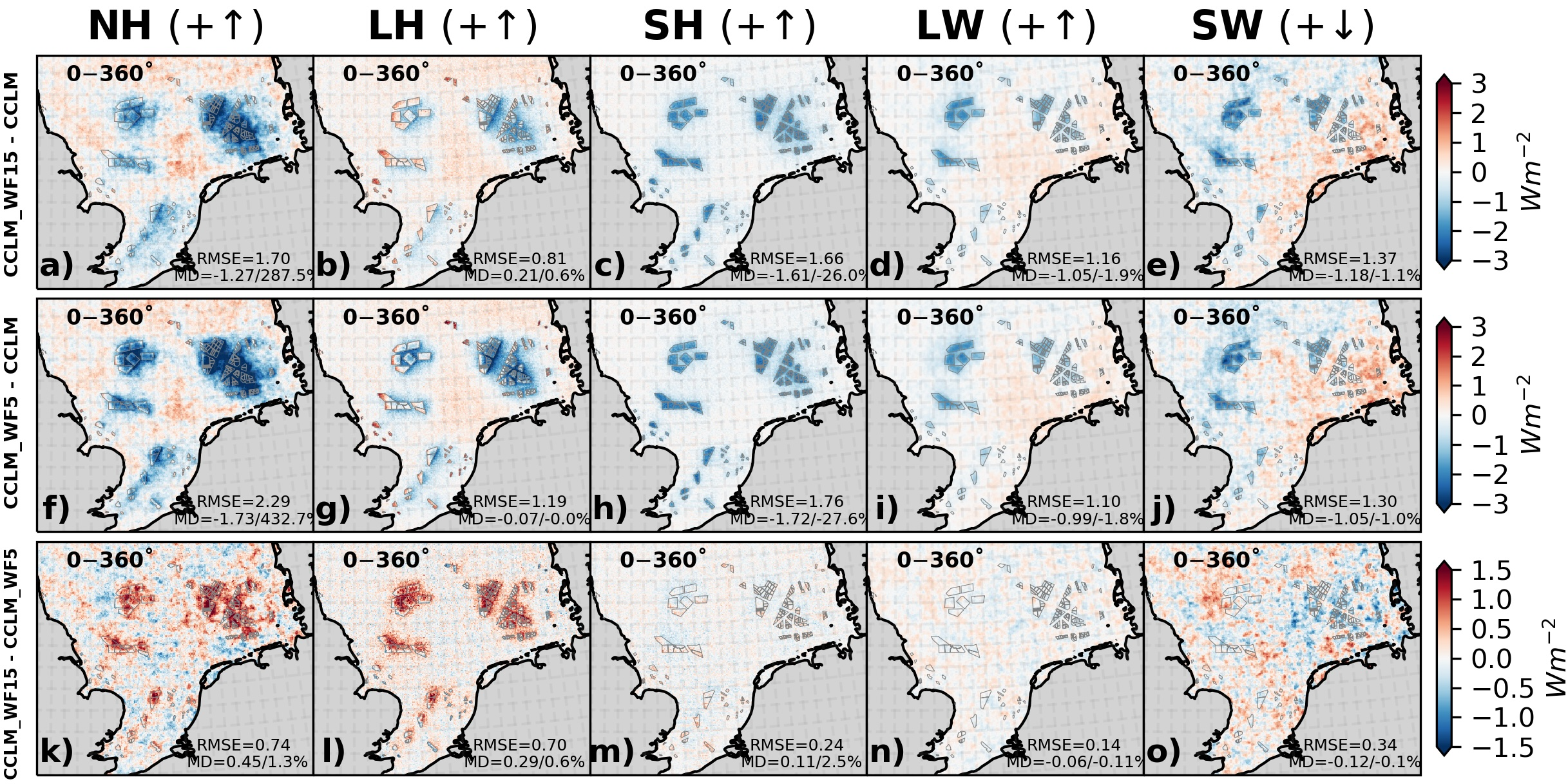


Fig.SI 6. Mean differences between CCLM_WF15 and CCLM (first row), CCLM_WF5 and CCLM (second row), and CCLM_WF15 and CCLM_WF5 (third row) for (fist column) net heat (NH) flux, (second column) latent heat (LH) flux, (third column) sensible heat (SH) flux, (fourth column) net upwelling longwave (LW) radiation, and (fifth column) net shortwave downwelling (SW) radiation, for wind directions of 0—360° during the period of 2008—2009. The legend provides Root Mean Square Errors (RMSE) and Mean Differences (MD) over the wind farm areas for the same period. This figure was created using Matplotlib (Hunter, J. D., Matplotlib: a 2D graphics environment. Computing in Science and Engineering 9, 2007) and Cartopy (Met office, Cartopy: a cartographic python library with a matplotlib interface. Exeter, Devon, https://scitools.org.uk/cartopy, 2015).

Fig. SI 7. The mean difference of low-level clouds between a) CCLM_WF15 and CCLM, b) CCLM_WF5 and CCLM, and c) CCLM_WF15 and CCLM_WF5 for wind directions (200—280˚) for the period of 2008—2009. The legend provides Root Mean Square Errors (RMSE) and Mean Differences (MD) over the wind farm areas for the same period. This figure was created using Matplotlib (Hunter, J. D., Matplotlib: a 2D graphics environment. Computing in Science and Engineering 9, 2007) and Cartopy (Met office, Cartopy: a cartographic python library with a matplotlib interface. Exeter, Devon, https://scitools.org.uk/cartopy, 2015).


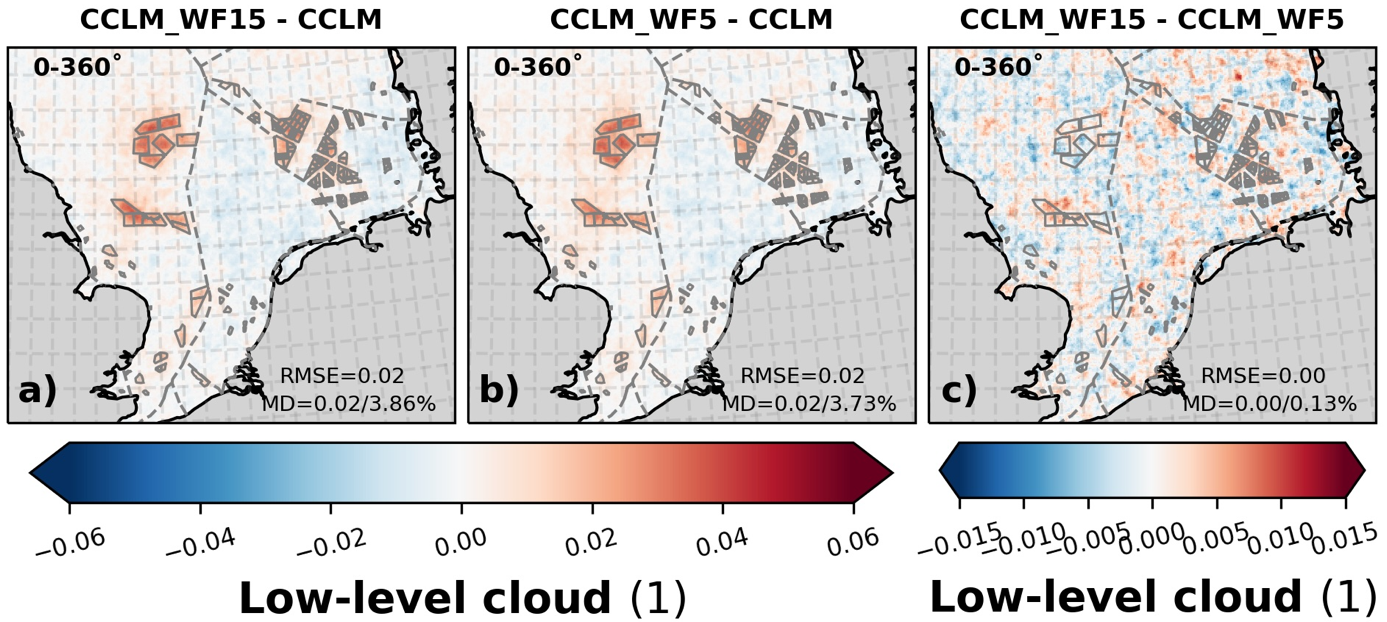


Fig. SI 8. The mean difference of low-level clouds between a) CCLM_WF15 and CCLM, b) CCLM_WF5 and CCLM, and c) CCLM_WF15 and CCLM_WF5 for all wind directions (0—360˚) for the period of 2008—2009. The legend provides Root Mean Square Errors (RMSE) and Mean Differences (MD) over the wind farm areas for the same period. This figure was created using Matplotlib (Hunter, J. D., Matplotlib: a 2D graphics environment. Computing in Science and Engineering 9, 2007) and Cartopy (Met office, Cartopy: a cartographic python library with a matplotlib interface. Exeter, Devon, https://scitools.org.uk/cartopy, 2015).


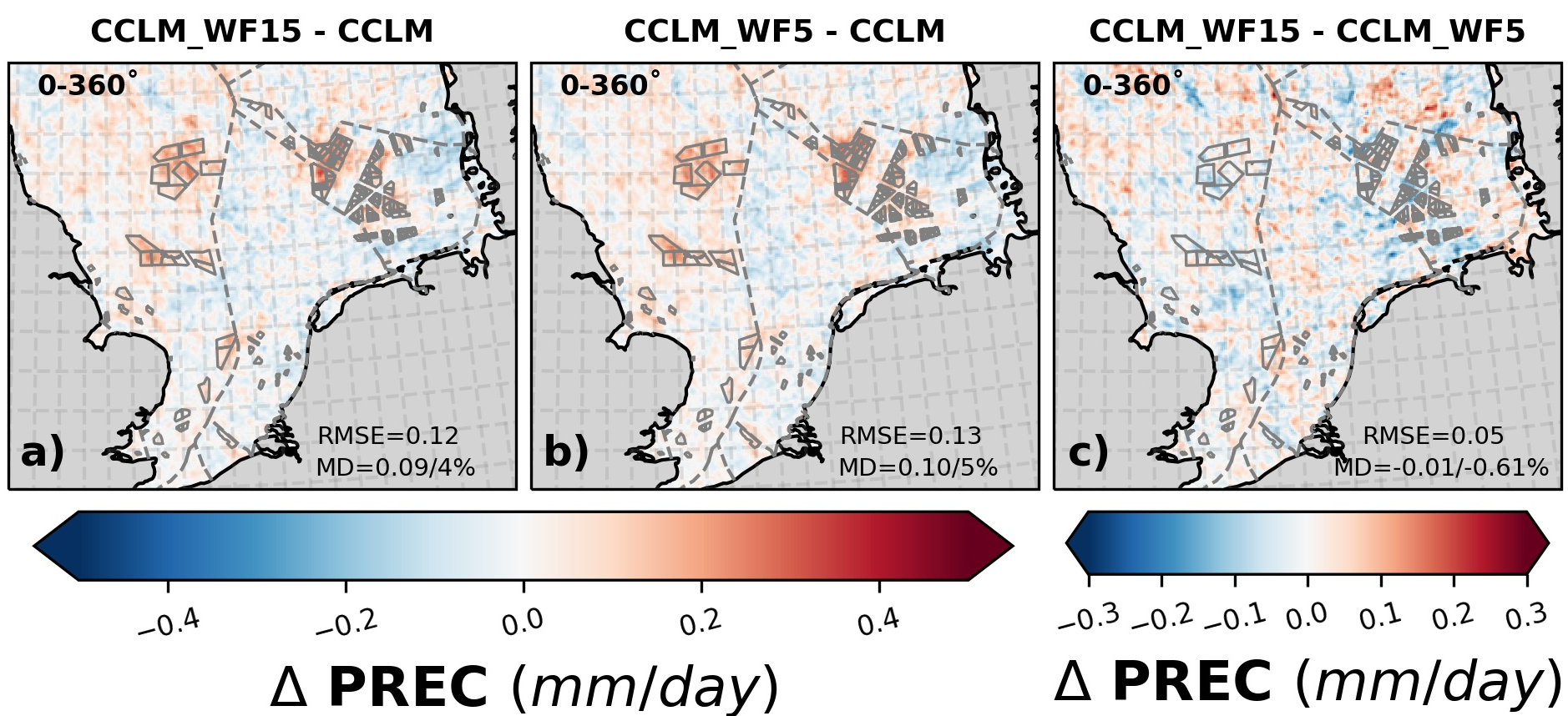


Fig. SI 9. The mean difference of precipitation (PREC) between a) CCLM_WF15 and CCLM, b) CCLM_WF5 and CCLM, and c) CCLM_WF15 and CCLM_WF5 for all wind directions (0—360˚) for the period of 2008—2009. The legend provides Root Mean Square Errors (RMSE) and Mean Differences (MD) over the wind farm areas for the same period. This figure was created using Matplotlib (Hunter, J. D., Matplotlib: a 2D graphics environment. Computing in Science and Engineering 9, 2007) and Cartopy (Met office, Cartopy: a cartographic python library with a matplotlib interface. Exeter, Devon, https://scitools.org.uk/cartopy, 2015).


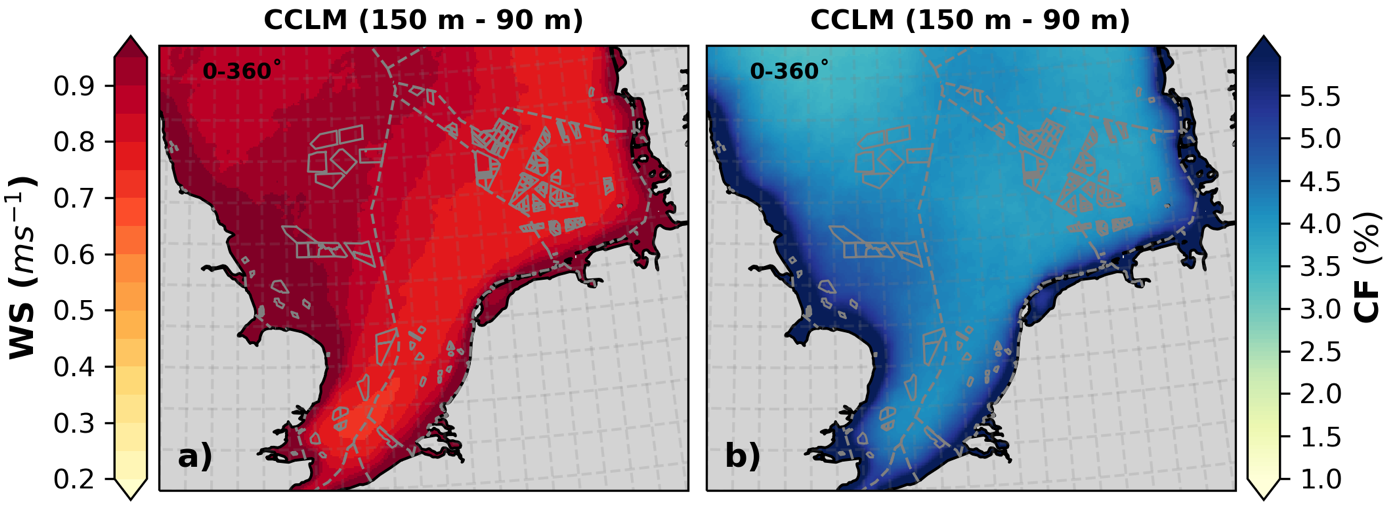


Fig.SI 10. This figure displays the annual mean difference between 150 m and 90m in a) wind speed (WS), and b) capacity factor (CF), for all wind directions (i.e., 0-360°) during the period of 2008—2009. This figure was created using Matplotlib (Hunter, J. D., Matplotlib: a 2D graphics environment. Computing in Science and Engineering 9, 2007) and Cartopy (Met office, Cartopy: a cartographic python library with a matplotlib interface. Exeter, Devon, https://scitools.org.uk/cartopy, 2015).


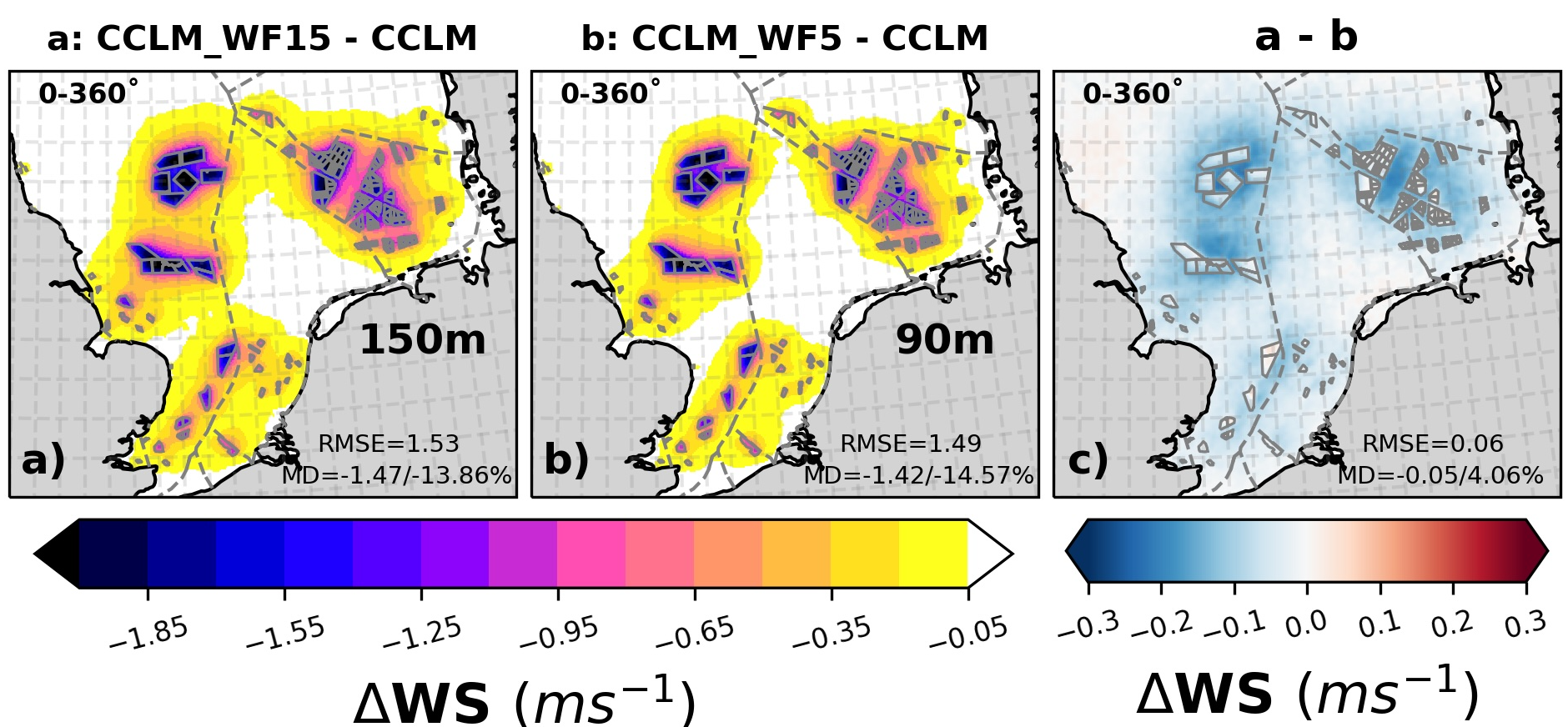


Fig. SI 11. The mean difference of wind speeds (WS) at hub height between a) CCLM_WF15 and CCLM (150 m), b) CCLM_WF5 and CCLM (90 m), and c) CCLM_WF15 and CCLM_WF5 for all wind directions (0—360˚) during the period of 2008—2009. The legend provides Root Mean Square Errors (RMSE) and Mean Differences (MD) over the wind farm areas for the same period. This figure was created using Matplotlib (Hunter, J. D., Matplotlib: a 2D graphics environment. Computing in Science and Engineering 9, 2007) and Cartopy (Met office, Cartopy: a cartographic python library with a matplotlib interface. Exeter, Devon, https://scitools.org.uk/cartopy, 2015).


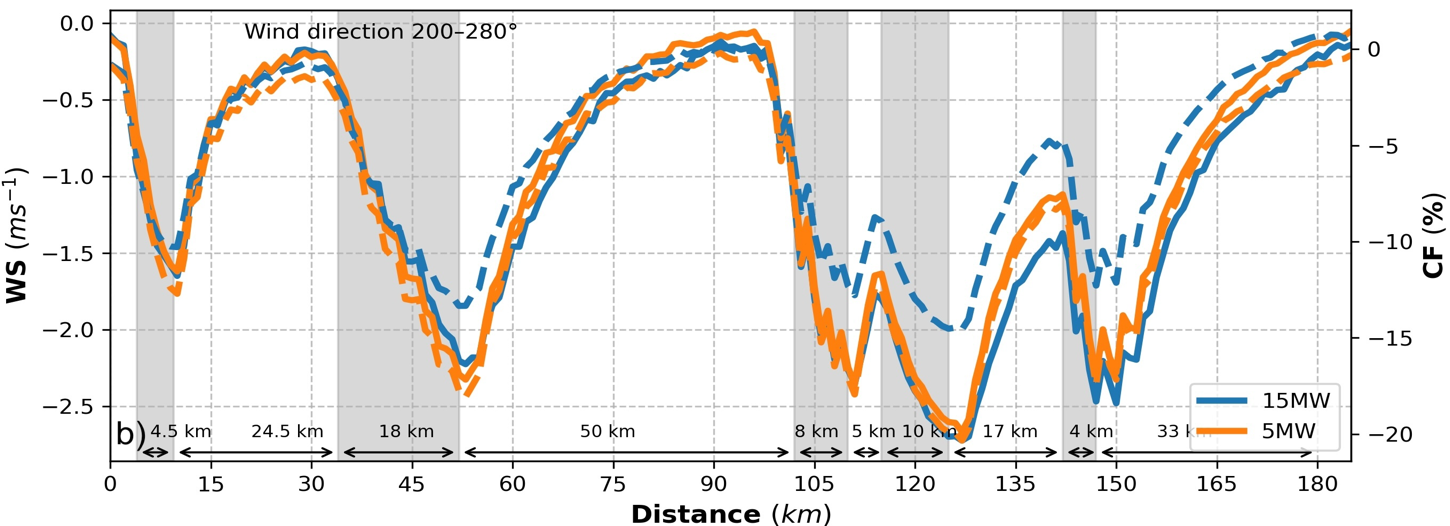

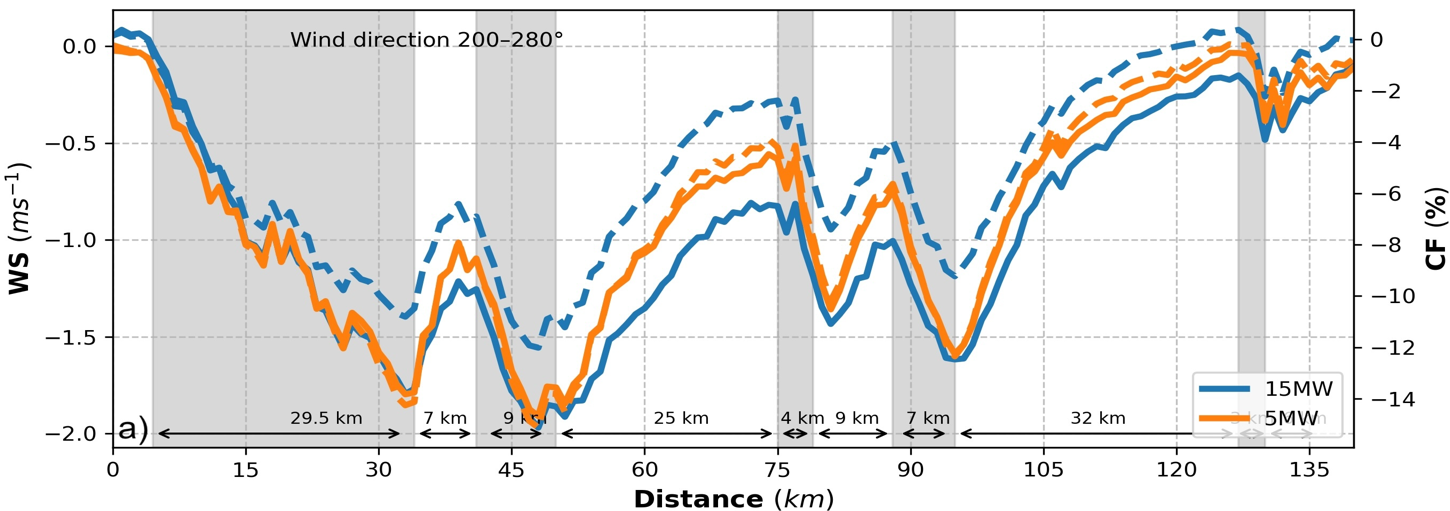

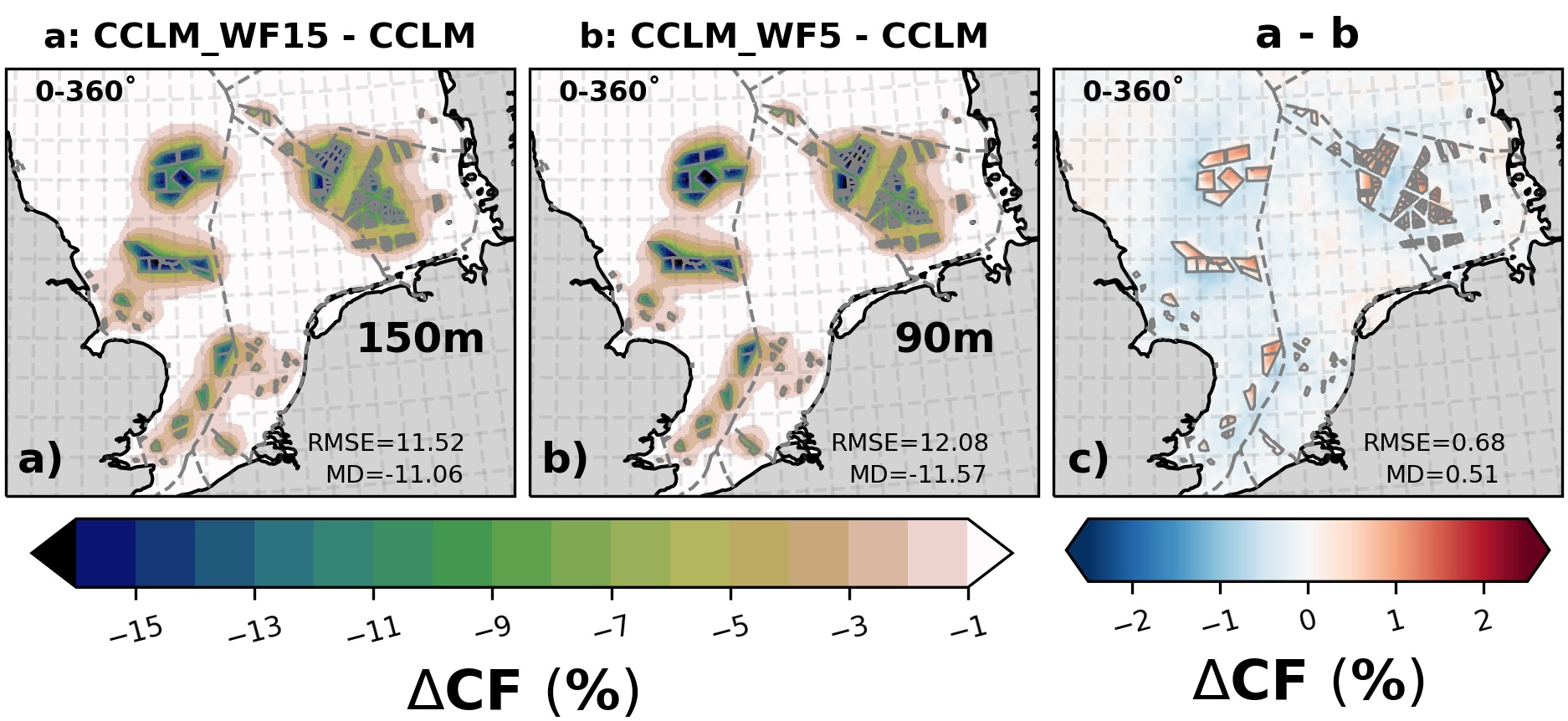


Fig. SI 12. The mean difference of capacity factor (CF) at hub height between a) CCLM_WF15 and CCLM, b) CCLM_WF5 and CCLM, and c) CCLM_WF15 and CCLM_WF5 for all wind directions (0—360˚) during the period of 2008—2009. The legend provides Root Mean Square Errors (RMSE) and Mean Differences (MD) over the wind farm areas for the same period. This figure was created using Matplotlib (Hunter, J. D., Matplotlib: a 2D graphics environment. Computing in Science and Engineering 9, 2007) and Cartopy (Met office, Cartopy: a cartographic python library with a matplotlib interface. Exeter, Devon, https://scitools.org.uk/cartopy, 2015).

*Fig. SI 13. Transects of wind speed (WS; left axis) and capacity factor (CF; right axis) deviation from mean wind speeds at hub height for the prevailing wind directions of 200–280° in 2008—2009 taken at a) transect I (position see Fig. SI 1) latitude 54.33°N–55.6°N and longitude 5.609°E–8.0°E, and b) transect III (see Fig. SI 1) latitude 53.4˚N—55.8˚N and longitude 0.8˚E—3.15˚E. Gray sectors indicate the wind farm positions.*
